# Wide-bandgap organic solar cells with a novel perylene-based non-fullerene acceptor enabling open-circuit voltages beyond 1.4 V†

**DOI:** 10.1039/d1ta09752k

**Published:** 2021-12-15

**Authors:** Jakob Hofinger, Stefan Weber, Felix Mayr, Anna Jodlbauer, Matiss Reinfelds, Thomas Rath, Gregor Trimmel, Markus C. Scharber

**Affiliations:** Linz Institute of Organic Solar Cells (LIOS), Institute of Physical Chemistry, Johannes Kepler University Linz Altenbergerstrasse 69 4040 Linz Austria jakob.hofinger@jku.at; Institute for Chemistry and Technology of Materials, NAWI Graz, Graz University of Technology Stremayrgasse 9 8010 Graz Austria; Institute of Applied Physics, Johannes Kepler University Linz Altenbergerstrasse 69 4040 Linz Austria

## Abstract

A perylene-based acceptor (PMI-FF-PMI), consisting of two perylene monoimide (PMI) units bridged with a dihydroindeno[1,2-*b*]fluorene molecule was developed as a potential non-fullerene acceptor (NFA) for organic solar cells (OSCs). The synthesized NFA was combined with the high-performance donor polymer D18 to fabricate efficient OSCs. With an effective bandgap of 2.02 eV, the D18:PMI-FF-PMI blend can be categorized as a wide-bandgap OSC and is an attractive candidate for application as a wide-bandgap sub-cell in all-organic triple-junction solar cell devices. Owing to their large effective bandgap, D18:PMI-FF-PMI solar cells are characterized by an extremely high open-circuit voltage (*V*_OC_) of 1.41 V, which to the best of our knowledge is the highest reported value for solution-processed OSCs so far. Despite the exceptionally high *V*_OC_ of this blend, a comparatively large non-radiative voltage loss (Δ*V*^non-rad^_OC_) of 0.25 V was derived from a detailed voltage loss analysis. Measurements of the electroluminescence quantum yield (ELQY) of the solar cell reveal high ELQY values of ∼0.1%, which contradicts the ELQY values derived from the non-radiative voltage loss (Δ*V*^non-rad^_OC_ = 0.25 V, ELQY = 0.0063%). This work should help to raise awareness that (especially for BHJ blends with small Δ_HOMO_ or Δ_LUMO_ offsets) the measured ELQY cannot be straightforwardly used to calculate the Δ*V*^non-rad^_OC_. To avoid any misinterpretation of the non-radiative voltage losses, the presented ELQY discrepancies for the D18:PMI-FF-PMI system should encourage OPV researchers to primarily rely on the Δ*V*^non-rad^_OC_ values derived from the presented voltage loss analysis based on EQE_PV_ and *J*–*V* measurements.

## Abstract 2

A novel wide-bandgap perylene-based acceptor (PMI-FF-PMI) allows the fabrication of efficient organic solar cells with an extraordinary high open-circuit voltage beyond 1.4 V.

## Graphical abstract

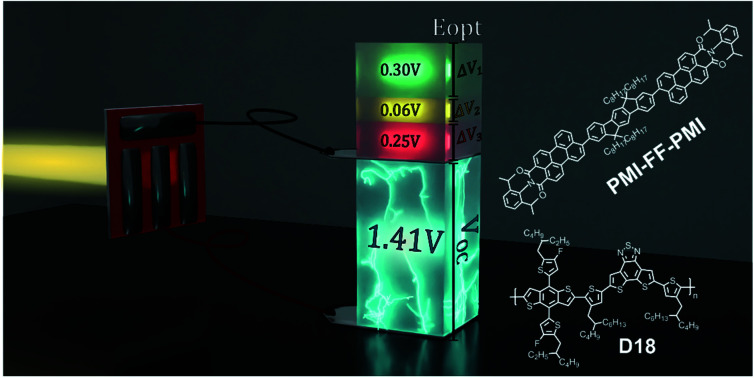

## 1. Introduction

During the last decade, the research field of organic photovoltaics (OPV) has witnessed the rise and prevalence of a new class of acceptor materials. The development of non-fullerene acceptors (NFAs) with superior light-harvesting properties and readily adjustable electronic energy levels compared to typical fullerene acceptors has led to a dramatic increase in power conversion efficiencies (PCEs) of bulk heterojunction (BHJ) organic solar cells (OSCs).^1,2^ In the last 5 years, PCEs of NFA-based solar cells have almost doubled and efficiencies of over 18% have been reported.^3–5^

Despite these rapid improvements, organic solar cell efficiencies still fall short compared to state-of-the-art inorganic or perovskite solar cells. As summarized in Table 1, the larger open-circuit voltage loss (Δ*V*^total^_OC_) of OPV devices can be identified as one of the main factors limiting the overall performance of organic solar cells. The total voltage loss of a solar cell is defined as the difference between the optical gap of the absorber (*E*_opt_) and the measured open-circuit voltage (*V*_OC_) under AM1.5G illumination as given by Δ*V*^total^_OC_ = (1/*q*)*E*_opt_ − *V*_OC_.

**Table 1:** Comparison of the photovoltaic parameters, open circuit voltage losses, and electroluminescence quantum yields (ELQYs) of selected organic, inorganic, and perovskite solar cells. *J*_SC_ and FF represent the short-circuit current density and the fill factor, respectively

Material	*E*_opt_ (eV)	*V*_OC_ (V)	*J*_SC_ (mA cm^−2^)	FF (%)	PCE (%)	Δ*V*^total^_OC_ (V)	Δ*V*^non-rad^_OC_ (V)	ELQY (%)	Source
GaAs	1.43	1.12	29.78	86.7	29.1	0.31	0.03	35.7	ref. 6 and 7
Perovskite	1.53	1.19	26.35	81.7	25.6	0.34	0.06	10.1	8
D18:Y6	1.38	0.87	25.24	73.6	16.1	0.51	0.20	0.04	9
D18:PC_71_BM	1.78	0.98	11.26	71.4	8.0	0.80	0.33	0.0003	9

Δ*V*^total^_OC_ can be expressed in terms of three individual voltage loss contributions as shown in eqn (1):1
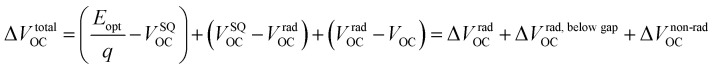


The three voltage loss terms in eqn (1) can be quantified using an analysis based on the following formula derived by Rau^10^2
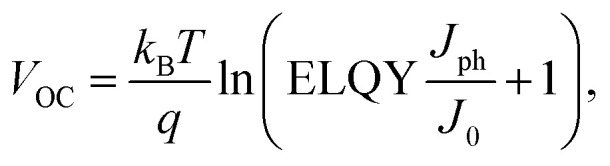
which allows the calculation of a solar cell's *V*_OC_ based on a detailed balance approach. In eqn (2), *k*_B_ is the Boltzmann constant, *T* is the temperature of the solar cell, *q* is the elementary charge, ELQY is the electroluminescence quantum yield, *J*_ph_ is the photocurrent and *J*_0_ is the dark saturation current. *J*_0_ and *J*_ph_ can be derived from sensitive EQE_PV_ measurements using the following equations:3
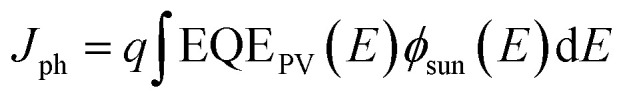
4
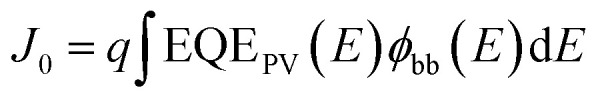


In eqn (3) and (4)*φ*_sun_ represents the AM1.5G solar spectrum, *φ*_bb_ is the black body spectrum at ambient temperature (300 K) and EQE_PV_ is the photovoltaic external quantum efficiency of the device. Assuming an ELQY equal to 1 allows the calculation of the *V*_OC_ in the radiative limit (*V*^rad^_OC_) for any solar cell device where the EQE_PV_ spectrum is known. Similarly, if the measured EQE_PV_ of the device in eqn (2) is replaced with an ideal step-function EQE_PV_, the *V*_OC_ in the Shockley–Queisser (SQ) limit (*V*^SQ^_OC_) can be calculated. When comparing the measured *V*_OC_ of a device to its *V*^rad^_OC_ or *V*^SQ^_OC_, the total voltage loss can be categorized into three different loss types according to eqn (1). A detailed description of the *V*_OC_ loss analysis is discussed in our recent publication, where we used the *V*_OC_ loss analysis to compare the non-radiative voltage losses of high-performance fullerene (D18:PC_71_BM) and non-fullerene (D18:Y6) solar cells.^9^ The results of the voltage loss analysis for the two organic solar cells are summarized in Table 1, revealing that the high-performance NFA-based solar cell exhibits significantly lower non-radiative voltage losses (∼0.2 V) compared to its fullerene-based counterpart (∼0.3 V). Nevertheless, the typically observed non-radiative voltage losses of high-performance NFA-based solar cells around 0.2 V are considerably larger than the 0.03 V or 0.06 V observed for top-end GaAs and perovskite solar cells, respectively. The formation of an interfacial CT state at the donor:acceptor (D/A) interface is thought to be responsible for the increased non-radiative voltage losses in OPV devices compared to their inorganic counterparts. Due to the low-dielectric constants of organic semiconductors and the resulting high exciton binding energies, a charge transfer (CT) state is required for the efficient dissociation of photogenerated excitons. Simultaneously, most of the recombination processes in OPV devices proceed *via* the CT state, underlining its importance for the performance of organic solar cells.

Recently, An *et al.*^11^ have reported an OPV device with an impressively low Δ*V*^non-rad^_OC_ of 0.16 V and an excellent electroluminescence quantum yield (ELQY) as high as 0.19%. The small voltage loss was ascribed to a high-lying CT state energy and a low offset (Δ*E*_LE-CT_) between the local exciton (LE) and CT state. It should be noted that for blends of a wide bandgap donor and small bandgap acceptor, the offset between the HOMO levels of donor and acceptor is usually taken as an estimate for the driving force to form a CT state and can be considered as a first approximation of the Δ*E*_LE-CT_ offset.^12^ Thus, the moderate observed EQE_PV_ values of around 40% reported by An and co-workers can be explained by the reduced driving force for CT state formation due to the small Δ*E*_LE-CT_ offset in this D/A blend. Amongst others,^13,14^ the work by An *et al.* highlights the inverse relationship between CT efficiency and non-radiative voltage loss in organic solar cells. Encouragingly, highly efficient polymer:NFA blends have recently been reported with a minimal energetic offset between the HOMOs of donor and acceptor.^15–17^ A better understanding of the high CT efficiencies despite the small energetic offsets in those OPV blends is required to develop new highly efficient D/A blends with minimal non-radiative voltage losses, closing the performance gap to efficient inorganic and perovskite solar cells.

A complementary approach to overcome the performance deficit caused by the large open-circuit voltage losses of OSC devices is to take advantage of the great variety and possibility to readily adjust the optical bandgaps of organic semiconductors. In addition, their solution processability allows easy fabrication of multi-junction devices consisting of stacked OPV blends with different optical bandgaps.

**Fig. 1:**
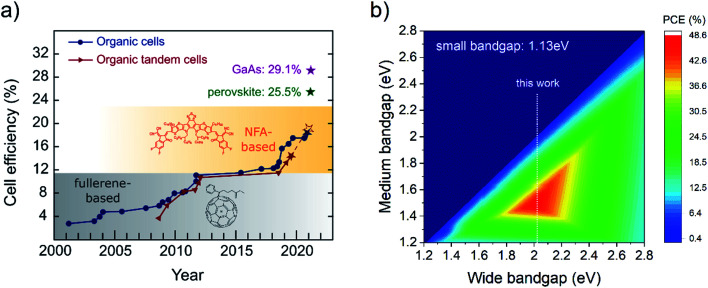
History of OSC efficiencies and triple-junction efficiency map. (a) Research cell efficiency chart for organic single-junction and tandem cells. Additionally, current record efficiencies for GaAs and perovskite solar cells are presented. The data was taken from the NREL efficiency chart.^18^ In addition, the 19.6% efficient tandem cell reported by Wang *et al.* is added to the NREL data and is depicted as a dashed star symbol.^5^ (b) 2D-efficiency map for a triple-junction device. The efficiencies were calculated in the SQ-limit for a fixed small bandgap absorber of 1.13 eV. The white dotted line represents the effective bandgap of the D18:PMI-FF-PMI blend.

According to the SQ-theory, only photons with energy larger than the bandgap get absorbed in an ideal single-junction solar cell. At short circuit current (*I*_SC_) conditions, the absorption is followed by a rapid thermalization to the bottom of the conduction band, where the exciton is separated into free charges. Photons with energy *E*_hν_ > *E*_opt_ lose the energy difference Δ*E* = *E*_hν_ − *E*_opt_ in form of heat during the thermalization process. In principle, a multi-junction device allows to significantly reduce thermalization losses due to a more efficient photon to energy conversion, enabling efficiencies well beyond the SQ-limit for single-junction devices of approximately 33%. However, efficiencies of tandem or triple-junction OPV devices based on fullerene acceptors seldomly exceeded those of single-junction devices as shown in Fig. 1a. In the past, the benefit of reduced thermalization losses in tandem devices was partly negated by the limited NIR absorption properties and the combined open circuit voltage losses of two fullerene-based sub-cells. As argued above state-of-the-art non-fullerene acceptors are primed to overcome both of these shortcomings. According to the NREL efficiency chart, the current record-breaking organic tandem device (PCE = 14.2%) still consists of a wide-bandgap, fullerene-based sub-cell.^18,19^ The development of wide-bandgap NFAs for applications in all-NFA-based tandem devices could reduce the overall voltage losses and significantly boost power conversion efficiencies, eventually fulfilling their promise in surpassing the efficiencies of single-junction devices. This statement has been confirmed by recent studies (not included in the current NREL efficiency chart^18^), claiming PCEs of almost 20% for OPV tandem cells with NFA-based wide-bandgap sub-cells.^5,20^

A study by Eperon *et al.*^21^ showed that the theoretical efficiency limit of triple junction devices is exceeding the efficiency limit of tandem devices only if spectral absorption around 1100 nm can be realized. The analysis is based on a simple SQ-model assuming a step-like EQE_PV_. For perovskite triple-junction cells the optimal bandgaps for wide, middle, and small-gap components derived from the simple SQ-model were confirmed (only minor shifts in optimal *E*_opt_ values) by a more realistic model based on a transfer matrix and device modeling approach. The good agreement between the two models highlights the validity of the simple approach in the framework of SQ-theory. Recently, an efficient organic solar cell based on a novel non-fullerene acceptor with strong infrared absorption up to 1100 nm (PTB7-Th:COTIC-4F) has been reported by Lee *et al.*^22^ As discussed above, optical absorption up to 1100 nm unlocks the regime, where the theoretical efficiency limit of triple junction devices is significantly increased compared to tandem devices. Moreover, other narrow-bandgap NFAs with absorption beyond 1000 nm have recently been reported such as SiOTIC–4F, CO1–4F and CO1–4Cl.^22–24^ Thus, the recent progress of low bandgap, non-fullerene acceptors strongly suggests developing specialized NFA-based organic solar cells optimized for usage in all-organic triple junction devices. A simple SQ-model, as discussed in ref. 21, was developed to identify the optimal bandgaps for an all-organic triple-junction device. Fig. 1b shows the maximum efficiency map in the detailed balance limit assuming a small bandgap absorber with a fixed bandgap of 1.13 eV (=1100 nm) while varying the bandgaps of medium and wide bandgap absorbers between 1.2 and 2.8 eV. A maximum theoretical efficiency of 48.6% was derived for a triple-junction device. Furthermore, Fig. 1b shows that wide and medium bandgap sub-cells with bandgaps in the range of 1.95–2.05 eV and 1.45–1.55 eV are required for optimal performance. A variety of high-efficient OSCs with an optical bandgap between 1.45 and 1.55 eV have already been reported.^25–29^ A well-established example is the D/A combination of PBDBT-2F (a.k.a. PM6) and IT-4F with a PCE and EQE_PV_ beyond 13% and 80%, respectively.^29^ On the contrary, high efficient OPV devices with an extremely low bandgap in the range of 1.1 eV are scarce. The development of novel NFAs like COTIC-4F is a promising start, but the currently only moderate EQE_PV_ values of around 50% obtained for ultra-small-bandgap OSCs suggest that further optimization is necessary to increase their performance.^22^ Similarly, due to the strong research effort of maximizing the absorption range of single-junction OPV blends, highly specialized wide-bandgap solar cells with effective bandgaps around 2 eV, required for triple-junction solar cell applications, are rare.

Herein, we report the synthesis, electrochemical and optical characterization of a wide-bandgap, perylene-based non-fullerene acceptor (PMI-FF-PMI). In combination with the commercially available, high-performance donor polymer D18, the newly developed acceptor is used to fabricate efficient, wide-bandgap, BHJ solar cells with extremely high *V*_OC_ values beyond 1.4 V. The photovoltaic parameters of the solar cells were investigated with *J*–*V*-response and EQE_PV_ measurements. Moreover, a detailed characterization of the electro- (EL) and photo luminescence (PL) properties of the D18:PMI-FF-PMI solar cells was performed. PL quenching experiments and time-correlated single-photon counting measurements (TCSPC) were conducted to analyze the driving force for CT state formation in this D/A blend. As discussed above, the high *V*_OC_ loss is one of the main limiting factors of OPV device efficiencies and furthering the understanding in this area is one of the main focal points of contemporary OPV research. Therefore, a D/A blend with a record-breaking high *V*_OC_ beyond 1.4 V is an interesting candidate to thoroughly investigate the individual open circuit voltage loss contributions using the presented voltage loss analysis. To evaluate the performance of the newly synthesized acceptor, the photovoltaic parameters and the determined voltage losses of D18:PMI-FF-PMI cells were compared to those of state-of-the-art fullerene (D18:PC_71_BM) and non-fullerene (D18:Y6) solar cells.^9^

## 2 Results

### 2.1 Experimental results

**Fig. 2:**
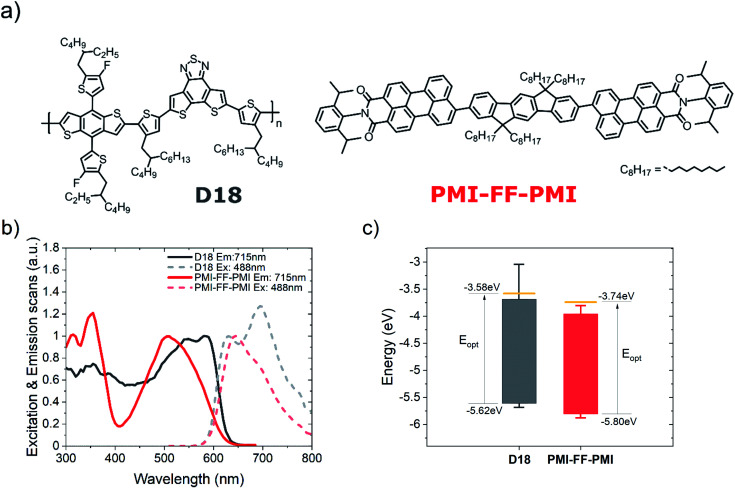
Chemical structure, optical and electrochemical characterizations. (a) Chemical structure of the D18 polymer and the PMI-FF-PMI small-molecule acceptor. (b) Measurements of the excitation and emission spectra of pristine D18 and PMI-FF-PMI. (c) HOMO and LUMO level estimations for the donor and acceptor materials obtained from electrochemical voltage spectroscopy measurements. The yellow line represents the theoretical LUMO_opt_ level calculated by adding *E*_opt_ to the HOMO level. The whiskers represent the maximum evaluation uncertainty of EVS measurements, as discussed in Note S2, ESI.†

In Fig. 2a, the chemical structures of the investigated OPV materials are presented. PMI-FF-PMI was synthesized *via* Suzuki coupling using a perylene pinacol ester and the linker dihydroindeno[1,2-*b*]fluorene dibromide. The detailed synthesis procedure is presented in Note S1 and Fig. S1, ESI.† The structure was verified by ^1^H and ^13^C APT NMR spectroscopy as well as MALDI-TOF mass spectrometry (see Fig. S2–S4, ESI†). The linker exhibits a larger conjugated π-system in the donor subunit compared to the recently investigated fluorene analog PMI-F-PMI.^30^ The newly synthesized acceptor PMI-FF-PMI shows strong optical absorption, good solubility in common chlorinated solvents, and excellent processability and film formation properties, which makes this molecule an interesting candidate to be tested in photovoltaic applications. Here, we investigated the performance of the perylene-based acceptor in combination with the commercially available donor polymer D18. A detailed optical characterization of D18 and PMI-FF-PMI in chlorobenzene solution is presented in Fig. S5, ESI.† The excitation and emission spectra of donor and acceptor thin films on glass are presented in Fig. 2b. The spectra are normalized to the low energy absorption and high energy emission peak, respectively. The PMI-FF-PMI absorption consists of two prominent peaks at 350 nm and 520 nm. The peak at 520 nm can be ascribed to the absorption of the PMI moieties, while the peak at 350 nm can be identified as the absorption of the dihydroindeno[1,2-*b*]fluorene linker. Furthermore, Fig. 2b displays a strong optical overlap of the excitation spectra of donor and acceptor. The crossing point of the excitation and emission spectra was used to estimate the optical bandgap.^31^ Both materials exhibit very similar wide optical bandgaps of 2.04 eV and 2.06 eV for D18 and PMI-FF-PMI, respectively. In addition, the absorption coefficients of D18, PMI-FF-PMI, and D18:PMI-FF-PMI (1 : 1) thin films are presented in Fig. S6, ESI.† Peak absorption coefficient values beyond 10^5^ cm^−1^ highlight the strong absorption properties of both materials.

In addition to the optical measurements, the OPV materials were electrochemically characterized using electrochemical voltage spectroscopy (EVS) and cyclic voltammetry (CV). During EVS measurements the system is kept close to its thermodynamic equilibrium. Thus, typical dynamic influences of standard CV measurements (*e.g.* scan speed) are reduced due to the slow, incremental variation of the applied potential. The HOMO and LUMO energy levels of the molecules presented in Fig. 2c were derived from EVS measurements of D18 and PMI-FF-PMI drop-casted thin films (see Fig. S7a, ESI†). A comparison between EVS and CV measurements is shown in Fig. S7b, ESI.† Following the evaluation procedure described in Note S2, ESI† results in HOMO energy levels of −5.62 eV and −5.80 eV and LUMO_opt_ (=HOMO + *E*_opt_) energy levels of −3.58 eV and −3.74 eV for D18 and PMI-FF-PMI, respectively. The EVS measurements indicate two large bandgap materials with similar energy levels. The system can be considered a small HOMO_D_-HOMO_A_ (Δ_HOMO_) and small LUMO_D_–LUMO_A_ (Δ_LUMO_) offset system with nominal offsets of 0.18 eV and 0.16 eV, respectively. As discussed in Note S2, ESI,† the presented values should be considered as rough estimates due to the large error margins of electrochemical measurements.^32^ However, the measurements were evaluated uniformly and the relative differences between the materials should thus provide a reliable insight into the relevant energy offsets of this D/A combination.

**Fig. 3:**
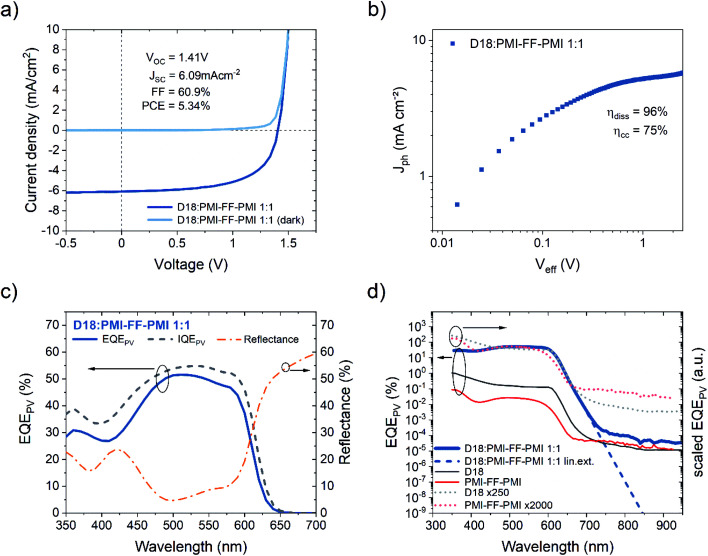
Photovoltaic characterization. (a) Current density–voltage curves in the dark (light blue) and under AM1.5G illumination (dark blue) of a D18:PMI-FF-PMI solar cell with a D/A ratio of 1 : 1. (b) Plot of the photogenerated current density (*J*_ph_) *vs.* the effective voltage (*V*_eff_). (c) High-sensitivity EQE_PV_ spectrum of a D18:PMI-FF-PMI solar cell on a linear scale (dark blue, solid line). Additionally, the reflectance (yellow, dash-dotted line) and the IQE_PV_ of the solar cell (grey, dashed line) are presented. (d) High-sensitivity EQE_PV_ spectra of a D18:PMI-FF-PMI solar cell (dark blue), a pristine D18 (black), and PMI-FF-PMI (red) diode on a semi-logarithmic scale (left axis). The dashed, dark blue line represents a “linear” fit of the EQE_PV_ in the region below the bandgap in the semi-log plot. The grey and red dotted lines illustrate the EQE_PV_ spectra of the pristine devices, which were scaled to match the EQE_PV_ of the D18:PMI-FF-PMI solar cell (right axis).

In the next step, the PMI-FF-PMI acceptor was used in combination with the donor polymer D18 to fabricate BHJ organic solar cells in a standard (glass/ITO/PEDOT:PSS/active layer/Ca/Al), as well as in an inverted (glass/ITO/ZnO/active layer/MoO_3_/Ag) device architecture. In the main text, we would like to focus on the devices fabricated in the standard configuration, while the results of the devices fabricated in the inverted configuration can be found in Fig. S8 and Table S1, ESI.† A detailed description of the device fabrication process is provided in the Methods section. Fig. 3a shows the characteristic *J*–*V* response of a D18:PMI-FF-PMI solar cell. The presented measurements were performed under AM1.5G (100 mW cm^−2^) illumination (dark blue curve) and in the dark (light blue curve) on a solar cell with a donor/acceptor ratio of 1 : 1. The derived photovoltaic parameters (*V*_OC_, *I*_SC_, FF, and PCE) of the *J*–*V* curve are summarized in Fig. 3a and Table 2. Additionally, the averages and standard deviations of 14 equivalent cells are presented in Table 2. As shown, D18:PMI-FF-PMI solar cells consistently exhibit extraordinary high *V*_OC_ values beyond 1.4 V. With *J*_SC_ values beyond 6 mA cm^−2^ and FFs of around 60%, the material combination allows for efficiencies over 5%. To the best of our knowledge, this D/A pair is the first system, based on organic semiconductors, to enable a *V*_OC_ beyond 1.4 V in combination with a PCE greater than 5%. In Fig. 3b the photogenerated current density (*J*_ph_) of the D18:PMI-FF-PMI solar cell is plotted *versus* the effective voltage (*V*_eff_). *J*_ph_ is defined as the difference between the current densities under AM1.5G illumination and in the dark (*J*_light_–*J*_dark_) and *V*_eff_ can be calculated by subtracting the series resistance corrected applied bias voltage (*V*_corr_ = *V*_app_ − *JR*_s_) from the voltage under *J*_ph_ = 0 conditions. From the ratio of *J*_ph_ at short circuit conditions and *J*_ph_ at *V*_eff_ = 2 V (=saturation current density *J*_sat_), the exciton dissociation efficiency (*η*_diss_) can be estimated. Similarly, the ratio of the current density at the maximum power point and the saturation current is used to estimate the charge collection efficiency (*η*_cc_).^33,34^ The derived values are presented in Fig. 3b. An exciton dissociation efficiency of 96% suggests that once a CT state is formed, the dissociation process into free charge carriers is highly efficient. Furthermore, a charge collection efficiency of 75% was derived. Light intensity-dependent measurements of the *J*–*V*-response of the D18:PMI-FF-PMI solar cells are presented in Fig. S9, ESI.†

**Table 2:** Summary of the photovoltaic parameters. Eqn (3) was used to calculate the short circuit current density (*J*_SC_EQE_) from the measured EQE_PV_ spectrum. Average values and standard deviations were calculated from 14 cells

Material	*V*_OC_ (V)	*J*_SC_ (mA cm^−2^)	*J*_SC_EQE_ (mA cm^−2^)	FF (%)	PCE (%)
D18:PMI-FF-PMI (ave.)	1.40 ± 0.01	6.11 ± 0.25	—	59.3 ± 1.0	5.12 ± 0.20
D18:PMI-FF-PMI (best)	1.41	6.09	6.04	60.9	5.34

The light-intensity dependence of the *J*_SC_ data is fitted to the power law 
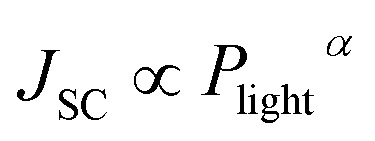
, where *P*_light_ is the average incident light intensity. An *α* value of 1 indicates negligible bimolecular recombination at *J*_SC_ conditions. Moreover, the ideality factor *n* was extracted from the light-intensity-dependent *V*_OC_ measurements using 
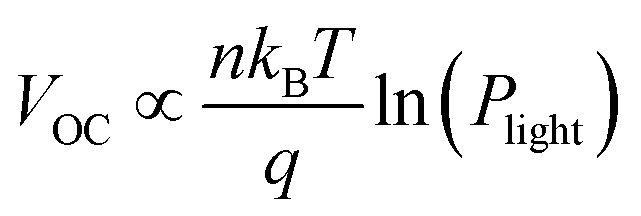
, where *k*_B_ is the Boltzmann constant, *T* is the temperature and *q* is the elementary charge.^35^ An ideality factor of 1.6 was derived for the D18:PMI-FF-PMI system, which is indicative of Shockley–Read–Hall recombination.

Moreover, in order to investigate the electronic transport properties of the novel PMI-FF-PMI acceptor, the electron mobility was determined from transfer characteristic measurements of organic field effect transistors (OFETs) in a bottom gate, top contact geometry as discussed in Note S3, ESI.† The measured transfer curves and the derived mobilities (*μ*^sat^_e_ = 1.3 × 10^−4^ cm^2^ V^−1^ s^−1^) are presented in Fig. S10a and b, ESI.† The reported hole mobility values of D18 range from 1.2 × 10^−3^ to 1.6 × 10^−3^ cm^2^ V^−1^ s^−1^, which suggest a slightly better hole than electron transport in D18:PMI-FF-PMI solar cells.^4,36^ However, the determined exponent *α* = 1 from light-intensity-dependent measurements suggests no severe imbalance of electron and hole mobilities in the optimized D18:PMI-FF-PMI device.^37^

In Fig. 3c the EQE_PV_ of a D18:PMI-FF-PMI solar cell is depicted. The EQE_PV_ curve exhibits spectral features of both donor and acceptor absorption and reaches its maximum value of 52% at 505 nm. As described in the Methods section, changing the angle of incidence of the monochromatic radiation in the EQE_PV_ experiment slightly (from 0° to 13°) allows measuring the reflected light intensity using a Si-photodiode. From this measurement, the spectral reflectance of the solar cell can be calculated, as shown in Fig. 3c (yellow, dash-dotted line). At maximum EQE_PV_ (505 nm), the reflectance reaches its minimum value of 4.7%, which mainly corresponds to the reflection at the glass substrate/air interface. Beyond 600 nm, the optical absorption of the active layer is severely reduced and the sharp increase of the reflectance can be related to the reflection at the highly reflective Ca/Al top electrode. Under the assumption that the Ca/Al electrode is a perfect mirror, and that all the non-reflected light gets absorbed by the active layer, it is possible to calculate the IQE_PV_ of the solar cell. The presented IQE_PV_ curve in Fig. 3c should be interpreted as a lower estimate of the actual IQE_PV_ spectrum since the underlying assumption neglects scattering, non-ideal reflection at the Ca/Al electrode, or parasitic absorption in the ITO or hole transport layer. In Fig. 3d the high-sensitivity EQE_PV_ spectrum, presented in Fig. 3c, is plotted on a semi-logarithmic scale. As depicted, the EQE_PV_ of the solar cell (dark blue curve) is recorded over a range of 6 orders of magnitude. EQE_PV_ values >700 nm are considered limited by the sensitivity of the measurement. As discussed in the introduction, the EQE_PV_ spectrum can be used to calculate the dark saturation current *J*_0_ using eqn (4). To avoid distortion of the determined *J*_0_ values by the measurement noise, a “linear” function (linear in the semi-log plot) was fitted to the tail of the EQE_PV_ (dark blue, dashed line), assuming that no physically relevant contributions to the EQE_PV_ spectrum are hidden below the sensitivity limit of the experimental setup. In addition, the EQE_PV_ spectra of the pristine D18 (black, solid line) and PMI-FF-PMI (red, solid line) devices are shown in Fig. 3d. The black and red dotted curves represent the EQE_PV_ spectra of the two pristine devices, each scaled with a constant factor to match the EQE_PV_ spectrum of the D18:PMI-FF-PMI solar cell. Comparing the scaled EQE_PV_ curves with the EQE_PV_ of the solar cell indicates that the sub-bandgap behavior of the solar cell is identical to the one of the pristine D18 device. This observation confirms that in this material blend the D18 donor polymer is the low-bandgap component and thus dictates the sub-bandgap behavior. Furthermore, the sub-bandgap behavior of the solar cell does not show any additional CT state absorption features, as often reported for fullerene-based solar cells.^9,38^

**Fig. 4:**
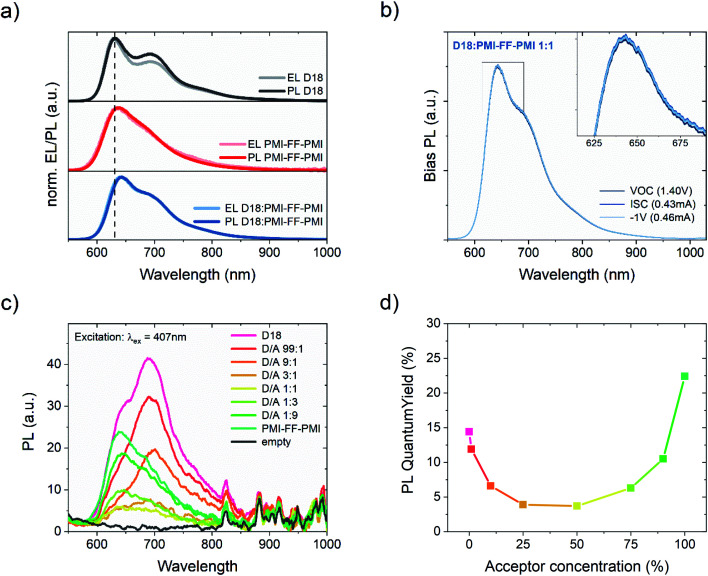
Photo- and electroluminescence measurements. (a) Normalized PL (excitation at 488 nm) and EL spectra of D18, PMI-FF-PMI, and the D18:PMI-FF-PMI blend with D/A ratio of 1 : 1. (b) Bias-dependent PL spectra of the D18:PMI-FF-PMI solar cell at *V*_OC_, *I*_SC_, −1 V. The inset shows an enlarged view of the PL maximum at 643 nm. (c) Integrating sphere PL measurements of D18, PMIFFPMI, and D18:PMI-FF-PMI thin films on glass with varying D/A ratios. The black curve represents a blank measurement without a sample inserted into the integrating sphere. An excitation wavelength *λ*_ex_ of 407 nm was used. (d) Absolute PLQY values as a function of acceptor concentration in the blend. The PLQY values were determined from the integrating sphere measurements presented in (c).

In addition to the photovoltaic characterization, the luminescence properties of the D/A blend and the pristine devices were investigated. The recorded photoluminescence (PL) and electroluminescence (EL) spectra are presented in Fig. 4a. A solid-state laser with an emission wavelength of 488 nm was used for photoexcitation. As discussed in the Methods section, the injection current during EL and the photocurrent during PL measurements were set to match the recorded *I*_SC_ under AM1.5G illumination. In the three panels of Fig. 4a, the normalized EL and PL spectra of the D18, PMI-FF-PMI, and D18:PMI-FF-PMI (1 : 1) devices are compared. The individual EL and PL spectra of all three devices exhibit similar emission features, which makes it difficult to analyze the spectrum of the D18:PMI-FF-PMI solar cell device. At first glance, the solar cell emission closely resembles the spectrum of the pristine D18 device. However, due to the strong spectral overlap of donor and acceptor emission, EL and PL measurements do not allow to exclude any emission contribution from the acceptor. Moreover, it should be emphasized that the low energy region of the EL spectrum of the D18:PMI-FF-PMI device does not show any sign of an additional CT state emission and closely resembles the EL spectrum of the pristine D18 device. In Fig. 4b bias-dependent photoluminescence measurements of a D18:PMI-FF-PMI solar cell are presented. In addition to the standard PL measurements at open-circuit conditions, the PL intensity was recorded under short-circuit (*I*_SC_) and reverse bias conditions (−1 V). The D18:PMI-FF-PMI solar cell does not show any signal reduction upon bias variation from *V*_OC_ to *I*_SC_. Even at an applied potential of −1 V, the PL signal intensity does not change. The bias-insensitivity of the PL signal suggests that the emission is mainly caused by radiative recombination of excitons, which are not involved in the charge generation and recombination processes in the solar cell and do not contribute to the photocurrent. In order to investigate the quenching efficiency in D18:PMI-FF-PMI blends, the absolute PLQY of D18:PMI-FF-PMI thin films on glass substrates with D/A ratios of 1 : 0, 99 : 1, 9 : 1, 3 : 1, 1 : 1, 1 : 3, 1 : 9, and 0 : 1 were measured with an integrating sphere setup. The individual PL spectra are presented in Fig. 4c and the extracted PLQY values as a function of acceptor concentration are presented in Fig. 4d. Measurements of the pristine D18 and PMI-FF-PMI films highlight the excellent emissive properties of both donor and acceptor. PLQY values of around 15% and 22% were observed for neat D18 and PMI-FF-PMI, respectively. Increasing the acceptor concentration to 1%, 10%, and 25% results in a continuous reduction of the PLQY. A further increase of the acceptor concentration to 75% or 90% leads to a rise of PLQY values, resulting in an overall u-shape as depicted in Fig. 4d. In a typical quenching experiment, the reduction of the PL intensity upon the introduction of a quencher is monitored. It is worth noting that due to the strong emissivity of the quencher (PMI-FF-PMI) and the strong spectral overlap of donor and acceptor emission (see Fig. 4c), the evaluation of the quenching efficiency is difficult. An increased acceptor concentration on the one hand quenches the polymer emission, but on the other hand, leads to a significant PL emission from the acceptor itself. However, for the D18:PMI-FF-PMI film with an optimized solar cell D/A ratio of 1 : 1, a mutual PL quenching is expected, where the emission of the donor is efficiently quenched by the presence of the acceptor and *vice versa*. The PLQY of the D18:PMI-FF-PMI 1 : 1 blend is reduced from 14.4% to 3.8% compared to the pristine D18 film.

**Fig. 5:**
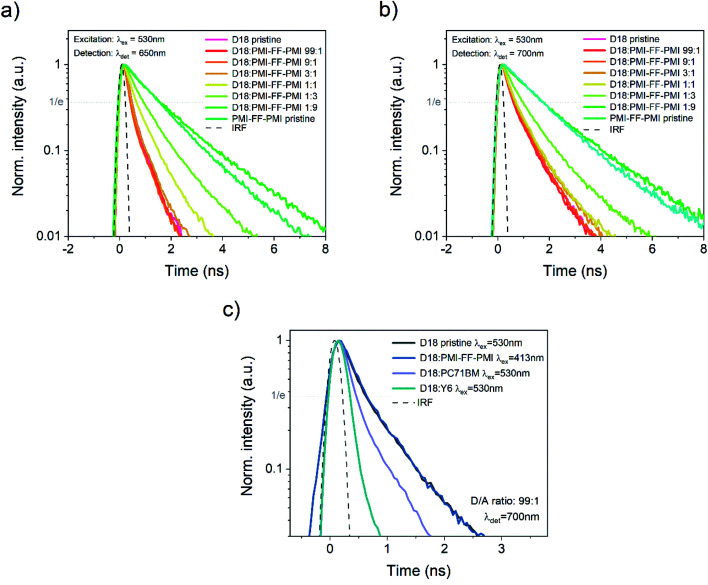
Time-correlated single-photon counting measurements. Time-resolved PL measurements of films of D18, PMI-FF-PMI, and D18:PMI-FF-PMI blends with varying D/A ratios. For all films, an excitation wavelength of 530 nm was chosen, while the detection wavelength was set to (a) 650 nm and (b) 700 nm. (c) Comparison of the time-resolved PL decay of pristine D18 *versus* D18:acceptor films with a D/A ratio of 99 : 1. The three materials PMI-FF-PMI, Y6, and PC_71_BM were used as acceptors. The excitation and detection wavelengths were set to 530 nm and 700 nm, respectively, except for the D18:PMI-FF-PMI (99 : 1) film where an excitation wavelength of 413 nm was used as rationalized in the text. In all three panels the instrument response function (IRF), which indicates the minimum time resolution of the experiment, is displayed as a black dashed curve. Additionally, the value where the normalized intensity is reduced to 1/*e* of its initial value is indicated by the grey dotted line. This value was used to compare the PL lifetimes.

In addition to the quenching experiments, time-correlated single-photon counting (TCSPC) measurements were performed to analyze the PL lifetimes of D18:PMI-FF-PMI blends with varying acceptor concentrations. TCSPC measurements of the thin films used in the PLQY quenching experiments (see Fig. 4c and d) are presented in Fig. 5a and b. For both measurements the excitation wavelength was set to 530 nm to ensure a balanced absorption between D18 and PMI-FF-PMI (see Fig. S6†). Based on the PL spectra presented in Fig. 4c, the detection wavelength was set to 650 nm (PMI-FF-PMI emission peak) and 700 nm (D18 emission peak), respectively. Both TCSPC measurements suggest that an increase of the acceptor concentration shifts the decay behavior from D18 dominated to PMI-FF-PMI dominated. The PL lifetimes of the blend films are found in between the lifetimes of pristine D18 and PMI-FF-PMI. A comparison of Fig. 5a and b indicates that the measured lifetime can be manipulated by changing the detection wavelength from 650 nm (Fig. 5a, pronounced PMI-FF-PMI emission) to 700 nm (Fig. 5b, pronounced D18 emission). The lifetime of the D18:PMI-FF-PMI 1 : 1 blend (yellow curve) in Fig. 5a is significantly increased compared to pristine D18 lifetime, while in Fig. 5b the lifetimes of the 1 : 1 blend and the pristine D18 film are almost identical. Fig. 5c shows the PL decay behavior of a D18:PMI-FF-PMI film with a D/A ratio of 99 : 1 in comparison to the behavior of analog D18 blends with state-of-the-art fullerene (PC_71_BM) and non-fullerene (Y6) acceptors. For the highly efficient D/A blends D18:Y6 and D18:PC_71_BM (EQE_PV_ > 80%), even a small amount of acceptor in the polymer film significantly reduces the observed PL lifetime as depicted in Fig. 5c. On the contrary, the decay curves of the pristine D18 film (black) and the D18:PMI-FF-PMI 99 : 1 film (dark blue) are congruent and no lifetime reduction can be observed.

**Fig. 6:**
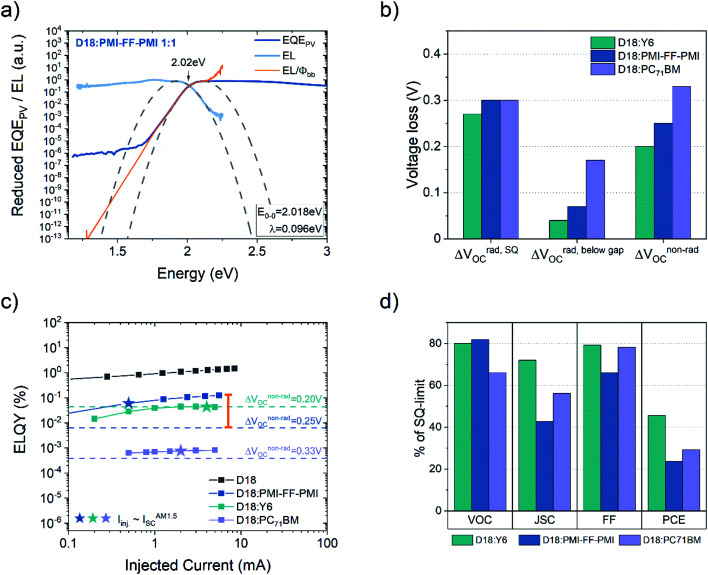
Analysis of the CT state energy, *V*_OC_ losses, ELQY, and photovoltaic parameters. (a) Reduced EL (light blue) and reduced EQE_PV_ (dark blue) spectra of the D18:PMI-FF-PMI solar cell. The dashed parabolas were calculated from eqn (8) and (9) using the derived fit parameters *E*_0-0_ and *λ*, which are presented in the small inset. (b) Comparison of the voltage losses derived for the D18:PMI-FF-PMI device with the ones reported for highly efficient non-fullerene (D18:Y6) and fullerene-based (D18:PC_71_BM) solar cells.^9^ The respective data is presented in Table 4. (c) ELQY measurements of D18:PMI-FF-PMI, D18:Y6, D18:PC_71_BM, and pristine D18 devices. The dashed lines represent the ELQY values calculated from eqn (10) assuming a non-radiative voltage loss of 0.20 V, 0.25 V, and 0.33 V for D18:Y6, D18:PMI-FF-PMI, and D18:PC_71_BM, respectively. The orange bar highlights the large discrepancy between the measured and calculated ELQY for D18:PMI-FF-PMI solar cells. (d) Illustration of the relative photovoltaic parameters of D18:Y6, D18:PMI-FF-PMI, and D18:PC_71_BM in % of their respective values in the SQ-limit.

In Fig. 6a, the energy difference between the relaxed ground and excited state (*E*_0-0_) of the D18:PMI-FF-PMI solar cell (2.02 eV) was determined from the crossing point of the reduced EQE_PV_ and the reduced EL spectrum.^31^ The orange curve represents the absorption spectrum of the D18:PMI-FF-PMI solar cell determined from its EL spectrum using the reciprocity relation EL/*φ*_bb_, where *φ*_bb_ is the black body radiation at 300 K. In Fig. 6b, the determined voltage loss contributions (see eqn (1)) of D18:PMI-FF-PMI are compared to the ones from high-performance D18 solar cells based on Y6 and PC_71_BM acceptors. The measured ELQY values of D18:PMI-FF-PMI, D18:Y6, D18:PC_71_BM, and pristine D18 devices are summarized in Fig. 6c. The star symbols highlight the situation where the injection current is equal to the short circuit current under AM1.5G illumination. Fig. 6d presents the photovoltaic parameters of the individual solar cells, normalized with respect to their maximum parameters in the Schockley–Queisser limit. The photovoltaic parameters in the SQ-limit were calculated assuming an ideal step function EQE_PV_^39^ and an optical gap of the small gap component of 1.36 (Y6), 2.02 (D18), and 1.78 eV (PC_71_BM).

## 3 Discussion

In the following, the photovoltaic parameters of D18:PMI-FF-PMI solar cells (see Fig. 3a) are compared to highly efficient solar cells based on D18 in combination with a fullerene (PC_71_BM) and non-fullerene acceptor (Y6). The two materials were chosen as examples of state-of-the-art fullerene and non-fullerene acceptors, which provide a good benchmark for the PMI-FF-PMI acceptor. D18:Y6 and D18:PC_71_BM solar cells are characterized by high EQE_PV_ values beyond 80%. Thus, optimizing the EQE_PV_ of the D18:PMI-FF-PMI blend (EQE_PV_ ∼ 50%) can be identified as one of the main challenges to close the performance gap to state-of-the-art fullerene, and non-fullerene-based solar cells. Therefore, in the next section, the presented experimental results are used to investigate the origin of the moderate EQE_PV_ values of D18:PMI-FF-PMI solar cells.

### 3.1 Charge generation efficiency in D18:PMI-FF-PMI blends

**Fig. 7:**
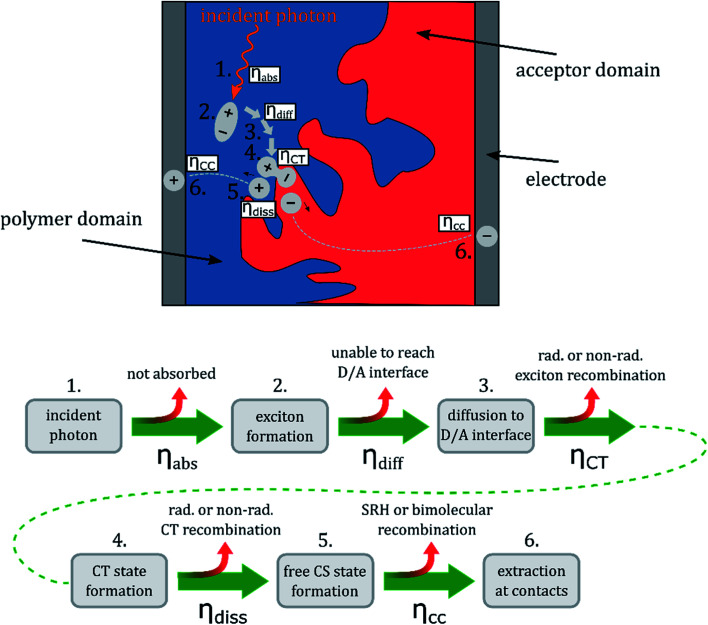
Photovoltaic conversion process of OSCs. Graphical and schematic illustration of the five efficiencies determining the EQE_PV_ of OSCs. It should be noted that the charge collection efficiency *η*_cc_ represents the combined efficiencies of charge transport and charge extraction. Common parasitic processes altering the respective efficiencies are indicated by the small red arrows.

As illustrated in Fig. 7, the EQE_PV_ of OSCs is determined by the efficiencies of five processes: light absorption (*η*_abs_), exciton diffusion to the D/A interface (*η*_diff_), CT state formation (*η*_CT_), CT dissociation into free charges (*η*_diss_), and charge collection (*η*_cc_). In principle, a shortcoming in one or more of the five efficiencies can cause the moderate EQE_PV_ values of D18:PMI-FF-PMI solar cells. The IQE_PV_ of the solar cell was estimated (see Fig. 3c) to exclude adverse effects on the EQE_PV_ due to limited photon absorption. The IQE_PV_ spectrum suggests that in the high absorption region of D18:PMI-FF-PMI, the EQE_PV_ is only slightly reduced due to reflection losses. At the maximum EQE_PV_, the reflectance of the solar cell is approaching the reflectance of the glass/air interface (∼4%), suggesting that almost all the photons are absorbed within the solar cell stack. As shown in Fig. 3b, the determined dissociation probability suggests that once a CT state is formed at a D18/PMI-FF-PMI interface, it has an extremely high probability of 96% to dissociate into free charges. With an estimated charge collection efficiency of 75%, none of the discussed processes is expected to severely limit the maximum EQE_PV_ value. Therefore, either a reduced driving force for CT state formation (low *η*_CT_) or a non-ideal blend morphology with D/A domain sizes exceeding the exciton diffusion length (low *η*_diff_) is expected to be responsible for the moderate EQE_PV_ values in D18:PMI-FF-PMI solar cells.

This hypothesis is in good agreement with the presented bias-dependent PL measurements in Fig. 4b. For an ideal PV device, the PL intensity is expected to vanish when the applied bias is swept from *V*_OC_ to *I*_SC_ conditions. At *V*_OC_ conditions the photogenerated charge carriers are forced to recombine radiatively within the device, resulting in a PL emission signal. At *I*_SC_ conditions, ideally, all photogenerated charge carriers are extracted at the contacts and charge carriers do not recombine in the active layer (no PL emission). Experimentally, a similar behavior has been reported for highly efficient perovskite solar cells (EQE_PV_ > 85%), where the PL intensity is strongly reduced when operated at *I*_SC_ conditions.^40^ With few exceptions, the PL of organic solar cells show very little or no bias-dependence.^41^ Typically, the small bias-dependent fraction of the PL signal is buried under a large bias-insensitive PL signal stemming from singlet emission of pristine donor or acceptor components. It should be noted that we recently have investigated the high-performance D/A system D18:Y6 (EQE_PV_ > 80%), which showed a moderate PL reduction of almost 10% when measured at *I*_SC_ conditions.^9^ Bias-dependent PL measurements of an efficient D18:Y6 solar cell are presented in Fig. S11, ESI.† Even the emission of this high-efficiency D/A blend primarily consists of a bias-insensitive component, which accounts for the majority (>90%) of the observed PL signal. However, the fact that there is a noticeable difference between *I*_SC_ and *V*_OC_ conditions is separating this system from most other D/A combinations and highlights its exceptional performance. In OPV blends the singlet emission can be caused by photovoltaically inactive, isolated pure donor or acceptor domains which can be a result of a non-ideal morphology. When the size of these domains exceeds the exciton diffusion length, the photogenerated excitons cannot diffuse to the D/A interface and are forced to recombine in the pure material. Even in the case of an ideal BHJ morphology with domain sizes smaller than the exciton diffusion length, the PL emission can be dominated by singlet exciton recombination if the driving force to form a CT state is weak. Thus, there is a direct competition between radiative and non-radiative exciton decay into the ground state and the formation of a CT state, if the rate for charge transfer is not significantly faster than the rate of singlet exciton recombination. Due to the typically high oscillator strength of singlet transitions in pristine donor or acceptor molecules, even a small fraction of recombining singlet excitons can overpower the weak emission from radiative CT state recombination. Therefore, the bias-insensitivity of the PL signal of D18:PMI-FF-PMI solar cells (see Fig. 4b) suggests that the recorded emission is dominated by radiative recombination of excitons, which do not contribute to the photocurrent. As discussed above, the photovoltaically inactive excitons are either caused by the presence of large domains of pristine material (non-ideal morphology) or a low charge transfer state efficiency.

Various attempts to change the D/A morphology by common processing techniques like (post-) annealing, solvent additives, solvent mixing, varying D/A ratios, or the processing temperature did not lead to a significant increase in the observed EQE_PV_ or *J*_SC_ values as summarized in Fig. S12, ESI.† Although non-ideal domain sizes cannot be excluded from the presented optimization trials, the inability to significantly improve the *J*_SC_ of the solar cells strongly suggests investigating the driving force of CT state formation. Especially, as electrochemical measurements of D18 and PMI-FF-PMI (see Fig. 2c) hint at small energetic offsets between the individual HOMO and LUMO levels of donor and acceptor. Atomic force microscopy (AFM) measurements did not allow to distinguish between donor and acceptor domains and could not be used to quantify the domain sizes.

A common way to investigate the charge generation efficiency in a D/A blend is to perform a PL quenching experiment.^13^ As shown in Fig. 4c and d, PLQY measurements were used to study the quenching of the PL intensity of D18 polymer thin films with increasing acceptor concentrations. The PLQY can be defined as the ratio of the radiative recombination rate (*k*_r_) to the sum of the rates of radiative (*k*_r_) and non-radiative (*k*_nr_) and the quenching rate (*k*_q_), as shown in eqn (5).^42^5
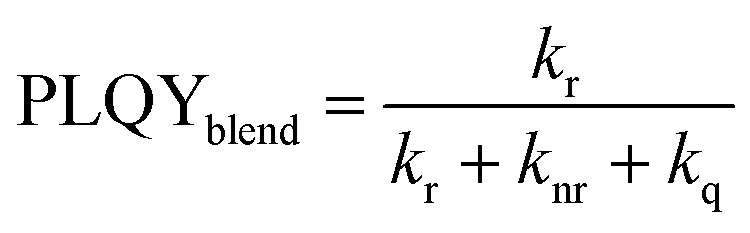
6
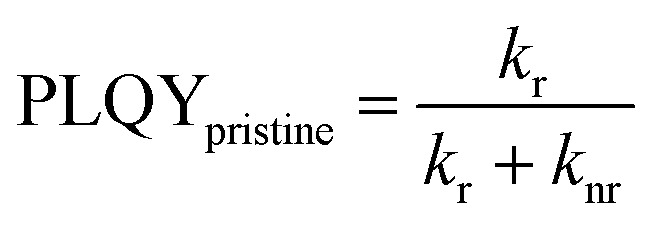
7
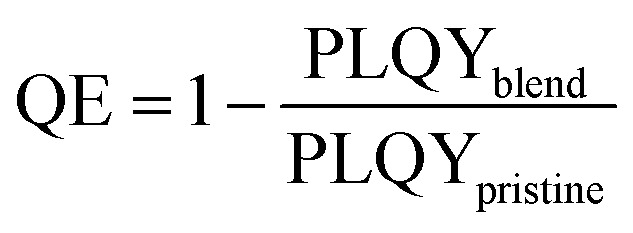


The quenching rate depends on the efficiency of the deactivation process of the excited state. In OPV blends quenching occurs when photogenerated excitons in a pristine domain can diffuse to a D/A interface and form a CT state. The radiative recombination rate of the CT state is typically several orders of magnitude lower compared to the one of the pristine materials and is thus assumed negligible. It should be stated that the quenching rate does not allow to differentiate between the effects of non-ideal morphology (*η*_diff_) or CT state formation efficiency (*η*_CT_). For pristine materials, the rate *k*_q_ is zero, and eqn (5) can be reduced to eqn (6). The quenching efficiency QE can be calculated according to eqn (7). It should be noted that the denominator in eqn (5) and (6) is dominated by the fastest rate. Fullerene-based solar cells are typically characterized by extremely fast CT rates and exhibit strong PL quenching efficiencies beyond 95%.^43^ In contrast, the quenching efficiency for the D18:PMI-FF-PMI film with a D/A ratio of 1 : 1 is around 74%. It has been shown that for a variety of different D/A pairs the quenching efficiency directly correlates with the maximum IQE_PV_ of the solar cell.^13^ For D18:PMI-FF-PMI this would lead to a maximum IQE_PV_ which is approximately 20% below the one from high efficient fullerene blends. This observation is in good agreement with the reduced EQE_PV_ of D18:PMI-FF-PMI solar cells (∼50%) compared to EQE_PV_ values >80% for D18:Y6 or D18:PC_71_BM solar cells. It should be noted that D18:PC_71_BM and D18:Y6 films with a D/A ratio of 99 : 1 showed a strong PLQY quenching below the detection limit of the integrating sphere setup. Assuming a PLQY detection limit of 1% allows estimating a lower limit for the quenching efficiency for D18:Y6 and D18:PC_71_BM thin films. From this estimation, the QEs of D18:Y6 and D18:PC_71_BM blends are expected to be larger than 93%. As discussed in Note S4, ESI,† PLQY and PL lifetime measurements (*τ*_meas_) of a pristine D18 film allow the calculation of the radiative (*k*_r_) and non-radiative (*k*_nr_) recombination rates in the pure polymer. Assuming that *k*_r_ and *k*_nr_ of the polymer in D/A blends are not affected by the presence of the acceptor allows determining the quenching rate *k*_q_ from PLQY measurements of the blends. Table 3 shows that a PLQY_blend_ of 11.9% for the D18:PMI-FF-PMI 99 : 1 blend yields a quenching rate of 3.4 × 10^8^ s^−1^, which is almost identical to the calculated radiative recombination rate (*k*_r_ = 2.3 × 10^8^ s^−1^). In contrast, the quenching rates of D18:Y6 and D18:PC_71_BM films with a D/A ratio of 99 : 1 are estimated to be beyond 2.2 × 10^10^ s^−1^, if a PLQY_blend_ < 1% is assumed. The film morphologies of all three 99 : 1 D/A blends are expected to be similar since the morphology is mainly determined by the D18 polymer. The unbalanced D/A ratio of 99 : 1 suggests large domains of pristine D18, while the small amount of acceptor is assumed to be evenly distributed in the polymer matrix. In this case, the diffusion of an exciton to the D/A interface (*η*_diff_) is assumed equally efficient for all three films. Consequently, the quenching rate *k*_q_ is directly related to the efficiency for CT state formation (*η*_CT_) and can be used to compare the driving force for charge transfer between the three D/A blends. The comparison between the quenching rates of D18:PMI-FF-PMI (∼2 × 10^8^ s^−1^) and D18:Y6 (∼2 × 10^10^ s^−1^) suggests that a reduced driving force for charge transfer can be identified as the main factor limiting the EQE_PV_ of D18:PMI-FF-PMI solar cells.

**Table 3:** PLQY and TCSPC lifetimes. PLQY and TCSPC measurements of D18 films with small amounts of PMI-FF-PMI, Y6, and PC_71_BM. The individual rates are calculated according to Note S4, ESI

D/A blend	D/A ratio	PLQY (%)	*τ*_meas_ (ns)	*k* ^calc^_r_ (s^−1^)	*k* ^calc^_nr_ (s^−1^)	*k* ^calc^_q_ (s^−1^)
Pristine D18	1 : 0	14.4	0.62	2.3 × 10^8^	1.4 × 10^9^	—
D18:PMI-FF-PMI	99 : 1	11.9	0.62	2.3 × 10^8^	1.4 × 10^9^	3.4 × 10^8^
D18:Y6	99 : 1	<1	0.35	2.3 × 10^8^	1.4 × 10^9^	>2.2 × 10^10^
D18:PC_71_BM	99 : 1	<1	0.45	2.3 × 10^8^	1.4 × 10^9^	>2.2 × 10^10^

A similar conclusion can be drawn from TCSPC measurements of the three 99 : 1 films as shown in Fig. 5c. The PL lifetime of a pristine D18 film can be identified as the denominator in eqn (5) with *k*_q_ = 0 (see eqn (S5), ESI†). Consequently, a quenching rate *k*_q_ which is in the same order of magnitude or larger than *k*_r_ and *k*_nr_ should lead to a significant reduction of the PL lifetime for D18 films with an acceptor concentration of 1%. Indeed, the measured PL lifetimes (*τ*_meas_) of high-performance D18:Y6 and D18:PC_71_BM blends are significantly lower (0.35 ns and 0.45 ns, respectively) compared to the lifetime of pristine D18 films (*τ*_meas_ = 0.62 ns). On the contrary, the lifetime of the D18:PMI-FF-PMI blend does not show any reduction and highlights the low quenching efficiency of this blend compared to efficient D18:Y6 or D18:PC_71_BM blends. Considering eqn (S5), ESI,† the lack of lifetime reduction upon the introduction of a quencher for the D18:PMI-FF-PMI blends (see Fig. 5a–c) seems counterintuitive. However, such a behavior can be rationalized by the strong emission contribution of pristine domains as discussed in Note S5, ESI.†

### 3.2 CT state & voltage loss analysis

In OSC blends the driving force for charge transfer is thought to be strongly related to the energetic offset between the local exciton (LE) state of the low bandgap component and the CT state (Δ*E*_LE-CT_). The proposed low driving force for CT state formation in D18:PMI-FF-PMI solar cells is in good agreement with the experimental results of EQE_PV_ and EL measurements. No clear evidence of CT state absorption or emission features can be extracted from the measurements of D18:PMI-FF-PMI solar cells (see Fig. 3d and 4a). To confirm these observations a common method suggested by Vandewal *et al.*^31^ was performed to determine the CT state energy. The method is based on the fact that transitions between the vibrationally relaxed ground and excited states (*E*_0-0_) can be described by mirror image Gaussian absorption *A*(*E*) and emission *N*(*E*) line shapes.8
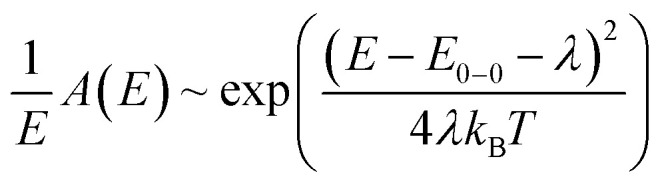
9
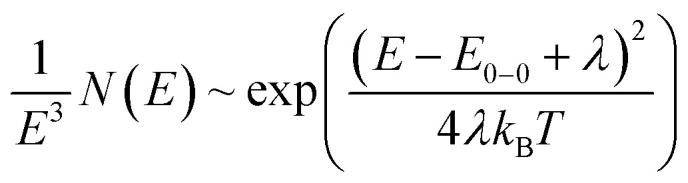


For OPV donor–acceptor blends the absorption *A*(*E*) and emission *N*(*E*) can be replaced with the measured EQE_PV_ and EL spectrum. Thus, the reduced absorption and emission spectra are obtained by dividing the measured EQE_PV_ and EL spectra by *E* and *E*^3^, respectively. In eqn (8) and (9)*E* represents the energy, *k*_B_ is the Boltzmann constant, *T* is the temperature and *λ* is the reorganization energy. For solar cells with a prominent CT state absorption and emission, the equations above can be used to fit the low energy CT absorption and high energy CT emission behavior as shown for D18:PC_71_BM.^9^ In this case the crossing point *E*_0-0_ can be identified as the CT state energy *E*_CT_. As shown in Fig. 6a the reduced emission spectrum of the D18:PMI-FF-PMI device does not show any sign of CT state emission. Therefore, eqn (9) was used to fit the high-energy part of the singlet emission peak. A two-parameter fit using a Levenberg–Marquardt iteration algorithm was performed. The final fit parameters *E*_0-0_ and *λ* are presented in the inset in Fig. 6a. The obtained fit parameters were used to construct the reduced absorption curve (see eqn (8) and (9)). Fig. 6a shows that the reduced absorption curve (dashed parabola) is in excellent agreement with the experimentally observed EQE_PV_ spectrum. Moreover, the reciprocity relation between the electroluminescence and the EQE_PV_ can be used to express the latter as the EL spectrum divided by the blackbody radiation at 300 K. The calculated EQE_PV_ spectrum using the reciprocity relation EL/*φ*_bb_ perfectly reproduces the experimentally observed EQE_PV_ behavior as shown in Fig. 6a. The analysis strongly highlights the importance of the D18 singlet absorption and emission properties, which completely dominate the EL and EQE_PV_ spectrum of D18:PMI-FF-PMI solar cells. In this case, the derived parameter *E*_0-0_ represents the optical bandgap of D18, and no exact value for the CT state energy can be derived from the performed measurements. The analysis suggests that potential CT state features are buried under the strong D18 singlet emission and absorption. Even if no exact value for the CT state energy could be determined, the lack of additional CT state absorption or emission features indicates that the CT state energy is not significantly lower than the singlet state energy of D18.

As discussed in the introduction, a better understanding of the voltage losses in OPV devices is of paramount importance for future device optimization. The extremely high *V*_OC_ beyond 1.4 V makes the D18:PMI-FF-PMI blend an interesting candidate to closely investigate the individual open circuit voltage loss contributions described in eqn (1). The EQE_PV_ measurements of the D18:PMI-FF-PMI device presented in Fig. 3c and d were used to perform the open-circuit voltage loss analysis as outlined in eqn (1)–(4). The results are summarized in Table 4. Despite the extremely high *V*_OC_ under AM1.5G illumination, the D18:PMI-FF-PMI solar cell exhibits a relatively high total open circuit voltage loss of 0.61 V. Furthermore, Table 4 shows the result of the voltage loss analysis for D18:Y6 and D18:PC_71_BM solar cells. The individual loss contributions of all three solar cells are summarized in Fig. 6b. The comparison of the voltage losses of solar cells based on the three different acceptors clearly shows that the PMI-FF-PMI device exhibits higher losses in all three categories compared to the highly efficient Y6-based device. Especially the non-radiative voltage loss of 0.25 V of the D18:PMI-FF-PMI solar cell is considerably higher than the 0.20 V of the D18:Y6 device. Still, the D18:PMI-FF-PMI device shows significantly less Δ*V*^rad,belowgap^_OC_ and Δ*V*^non-rad^_OC_ losses compared to the fullerene-based device. The results confirm the frequently observed trend that NFA-based solar cells suffer from less open circuit voltage losses than their fullerene-based counterparts.

**Table 4:** Comparison of the voltage losses in D18 solar cells with different acceptor molecules

Material D18	*E*_opt_/*q* (V)	Δ*V*^rad,SQ^_OC_ (V)	*V* ^SQ^_OC_ (V)	Δ*V*^rad,b.g.^_OC_ (V)	*V* ^rad^_OC_ (V)	Δ*V*^non-rad^_OC_ (V)	*V*_OC_ (V)	Δ*V*^total^_OC_ (V)	Source
PMI-FF-PMI	**2.02**	0.30	**1.72**	0.06	**1.66**	0.25	**1.41**	0.61	This work
Y6	**1.38**	0.27	**1.11**	0.04	**1.07**	0.20	**0.87**	0.51	9
PC_71_BM	**1.78**	0.30	**1.48**	0.17	**1.31**	0.33	**0.98**	0.80	9

As a direct consequence of eqn (2), the non-radiative voltage loss can be directly linked to the ELQY of a solar cell device according to10
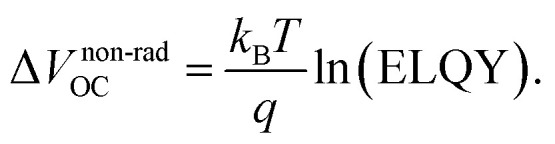


Therefore, additional measurements of the ELQY of the solar cell can be used to validate the performed voltage loss analysis. As shown in Fig. 6c, the calculated ELQYs (ELQY_calc_) from non-radiative voltage losses (dashed lines) are in good agreement with the measured ELQY (ELQY_meas_) for D18:Y6 and D18:PC_71_BM solar cells. As highlighted by the orange bar in Fig. 6c, the ELQY_meas_ values of the D18:PMI-FF-PMI solar cell are approximately a factor of ten higher than the ELQY calculated from the observed non-radiative voltage loss of 0.25 V. ELQY_meas_ values as high as 0.1% were recorded for the D18:PMI-FF-PMI device, which would correspond to a non-radiative voltage loss of only 0.18 V. The *V*_OC_ of such devices should be as high as 1.48 V, which is contradictory to the experimentally observed *V*_OC_ values. The large discrepancy between the measured and calculated ELQY is further discussed in the next section.

In addition to the voltage losses, the photovoltaic performance of D18:PMI-FF-PMI, D18:PC_71_BM, and D18:Y6 solar cells was compared. Due to the different effective bandgaps of the three solar cells, the measured photovoltaic parameters were normalized by the respective photovoltaic parameters in the SQ-limit. With the knowledge of the effective bandgap of the blends and assuming an ideal step-like EQE_PV_, *J*^SQ^_0_ and *J*^SQ^_SC_ can be calculated using eqn (3) and (4). The derived values can be used to construct the *J*–*V* curve in the SQ-limit using the following formula.11
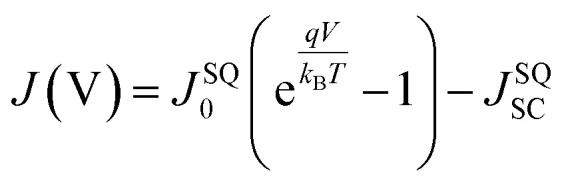


The photovoltaic parameters in the SQ-limit can then be extracted from the obtained *J*–*V*-curves. The measured photovoltaic parameters and calculated SQ-photovoltaic parameters are summarized in Table S2, ESI.† In addition, the measured photovoltaic parameters of all three solar cells in percent of their respective SQ-limit are graphically illustrated in Fig. 6d. The *V*_OC_ of the D18:PMI-FF-PMI cell reaches an impressive value of 82% of its maximum value, which once again highlights the extraordinary high *V*_OC_ in this D/A blend. Moreover, it can be seen that the overall performance of this blend is limited by the moderate *J*_SC_ values. Only 42% of the ideal *J*_SC_ in the SQ-limit is reached, which is significantly lower than the 72% of the highly efficient D18:Y6 system. Once again, the moderate EQE_PV_ values of the D18:PMI-FF-PMI solar cell can be identified as the main factor limiting the overall photovoltaic performance. In addition, Fig. 6d shows that the D18:PMI-FF-PMI device reaches only 66% of its FF^SQ^ compared to approximately 80% for D18:Y6 and D18:PC_71_BM, indicating another possibility to further optimize the device performance.

### 3.3 ELQY *vs.* non-radiative voltage loss

To elucidate the large differences between ELQY_meas_ and ELQY_calc_, the non-radiative voltage losses and electroluminescence properties of D18:PMI-FF-PMI solar cells are discussed within the framework of a three-state-model reported by Chen and co-workers, which specifically incorporates the interaction (hybridization) between LE and CT states.^12^ Their model accounts for the thermal population of LE and CT states and can be used to determine the radiative and non-radiative recombination rates of OPV devices. With the extended three-state model they are able to explain the energy gap law dependence found in fullerene-based blends,^44^ as well as the deviation from the energy gap law in state-of-the-art NFA-based blends. The three-state model highlights the importance of the Δ*E*_LE-CT_ offset and the electronic coupling between LE and CT state (*t*_LE-CT_). Moreover, they show that for low Δ*E*_LE-CT_ offset systems the ELQY approaches the ELQY of the pristine low bandgap component, which is ultimately determining the minimum non-radiative voltage loss for any D/A blend. Within the framework of the energy gap law and the three-state model, the D18:PMI-FF-PMI solar cell is expected to exhibit minimal non-radiative voltage losses. As discussed above, the presented experimental data show no clear CT state absorption or emission features and suggest that the CT state is energetically close to the LE state of D18 (∼2 eV). The assumption of a small Δ*E*_LE-CT_ offset system is supported by the low quenching efficiency and the moderate EQE_PV_ values of D18:PMI-FF-PMI solar cells. According to the energy gap law, the high CT state energy should lead to a reduced wavefunction overlap between the relaxed CT state and higher-order vibrational modes of the ground state. Moreover, as suggested by the three-state model, the D18:PMI-FF-PMI blend should benefit from LE-CT state hybridization due to a low Δ*E*_LE-CT_ offset. Both of these properties hint at a D/A blend with an extremely low non-radiative voltage loss. However, these predictions cannot be confirmed with the presented voltage loss analysis, as a moderate Δ*V*_OC_^non-rad^ loss of 0.25 V, corresponding to an ELQY_calc_ of 6.4 × 10^−3^%, was observed. As highlighted in Fig. 6c, there exists a vast discrepancy between ELQY_calc_ and the measured ELQY of the solar cell (1.2 × 10^−1^%). It should be noted that the ELQY of the D18:PMI-FF-PMI solar cell is indeed approaching the ELQY of the pristine D18 device as predicted by the three-state model. However, the high measured ELQY values do not translate to a reduced non-radiative voltage loss of the solar cell device.

In the following, we will argue that the measured ELQY value is overestimating the radiative efficiency of the solar cell. From PLQY quenching and bias-dependent PL experiments we have derived that the PLQY of a D18:PMI-FF-PMI blend with a D/A ratio of 1 : 1 is approximately 3%, and we have observed that the emission spectrum of the solar cell is independent of the applied bias. At *V*_OC_ conditions all the photogenerated free charge carriers are forced to recombine within the active layer, while at *I*_SC_ conditions the PL intensity should be reduced due to the extraction of these free charge carriers. The minimal detectable change in PL intensity of the experimental setup is assumed to be 1%. Considering the invariance of the PL intensity upon the applied bias leads to the conclusion that the additional emission from radiative recombination of free charge carriers accounts for less than 1% of the total emission. Thus, the ELQY of free charge carrier recombination in the D18:PMI-FF-PMI blend has to be lower than 3 × 10^−2^%. The derived ELQY value from the voltage loss analysis (ELQY_calc_ = 6.4 × 10^−3^%.) is consistent with the derived upper limit of 3 × 10^−2^%. On the contrary, recombination of injected free charge carriers can be ruled out as an origin of the experimentally measured, large ELQY values (ELQY_meas_ = 1.2 × 10^−1^%), since such a large free-carrier contribution would lead to a significant bias-sensitive component in the bias-dependent PL measurement. As clearly shown in Fig. 4b, a bias dependency of the PL signal is not observed for the D18:PMI-FF-PMI device.

However, the source of the additional EL emission contribution, leading to an overestimation of the measured ELQY, remains unclear. A possible explanation for the increased ELQY is the charge injection into pure donor or acceptor domains. Especially for large applied voltages, the electrical energy of injected charge carriers might be high enough to elevate an electron into the LUMO level of the polymer or a hole into the HOMO level of the acceptor, owing to the small energetic offsets between D18 and PMI-FF-PMI. Subsequent recombination of an electron and a hole on the pristine material would be able to explain the boosted ELQY of the solar cell due to the typically larger ELQYs of pristine devices. A schematic sketch of the band diagram of D18:PMI-FF-PMI depicts the electron injection from a metal electrode at different forward bias conditions and highlights the possibility of direct electron injection into the polymer for an OPV blend with small LUMO offsets (see Fig. S13a, ESI†). Moreover, Fig. S13b, ESI† shows that the pristine D18 device already exhibits high ELQY values at applied voltages between 1.4–1.5 V (close to *V*_OC_ of the solar cell). Thus, even at moderate applied voltages, an efficient electron injection in the D18 device is expected. It should be noted that also the reverse process of injecting a hole into the HOMO of the acceptor molecule cannot be excluded. This process is conceivable for all small Δ_HOMO_ or Δ_LUMO_ offset systems and could in principle affect ELQY measurements of most common wide-bandgap donor, small bandgap acceptor systems (*e.g.* OSCs based on the Y-acceptor series).

In addition to the effect of direct charge injection, the measured ELQY can be strongly affected by the applied bias voltage, charge carrier mobility, and injection barriers. Regardless of these challenges, various reports have shown excellent agreement between ELQY_calc_ and ELQY_meas_, supporting the feasibility of deriving the Δ*V*^non-rad^_OC_ loss of a solar cell from ELQY measurements.^12,45^ However, our detailed study of the emission properties of D18:PMI-FF-PMI solar cells indicates that this is not generally true. Our results suggest that there are exceptions, where the measured ELQY is massively overestimating the ELQY derived from the Δ*V*^non-rad^_OC_ losses of the solar cell. Despite being common practice to determine the Δ*V*^non-rad^_OC_ loss from ELQY measurements, our results strongly emphasize that solely relying on this method is precarious and that the derived Δ*V*^non-rad^_OC_ values should always be validated by performing a voltage loss analysis as described earlier.

### 3.4 Application in triple junction devices & optimization potential

The recent success of NFA-based tandem devices highlights the great potential of increasing OPV efficiencies by a more efficient photon-to-energy conversion due to reduced thermalization losses in multi-junction OPV devices. As discussed in the introduction, the promising development of ultra-low bandgap OPV blends with strong infrared absorption up to 1100 nm unlocks the possibility of efficient triple-junction OPV stacks. With an effective bandgap of around 2.02 eV, the D18:PMI-FF-PMI device can be identified as an ideal candidate for a wide-bandgap sub-cell in all-organic triple-junction devices. The impressive *V*_OC_ of 1.41 V for the D18:PMI-FF-PMI system suggests triple-junction open-circuit voltages close to 3 V, as theoretically shown in Fig. S14, ESI.† Here, the OPV blends D18:PMI-FF-PMI, PBDBT-2F:IT-4F, and PTB7-Th:COTIC-4F are presented as possible candidates for an all-organic triple-junction device. The simple model described in Note S6, ESI† predicts that triple junction efficiencies around 15% and 20% can be realized if the maximum EQE_PV_ of the wide-bandgap (D18:PMI-FF-PMI) and small-bandgap sub-cell (PTB7-Th:COTIC-4F) can be improved to 70% and 85%, respectively. Thus, in order to fabricate efficient triple-junction devices, further device and material optimizations are necessary to improve the EQE_PV_ of D18:PMI-FF-PMI solar cells. The presented experimental results indicate that a slight modification of the PMI-FF-PMI molecule to increase the Δ_HOMO_ or Δ_LUMO_ offset between donor and acceptor could be beneficial for the charge generation efficiency. An often-used approach to slightly reduce the energy levels of NFAs is halogenation (*e.g.* chlorination or fluorination). Chemical modification of the perylene π-system by functionalization at the bay positions^46^ could be a feasible strategy to further improve the EQE_PV_ of D18:PMI-FF-PMI solar cells. Alternatively, it could be beneficial to increase the bandgap of the acceptor (ideally without increasing its LUMO). A larger bandgap would lead to strong complementary absorption in the UV – blue region of the solar spectrum in addition to the strong D18 absorption between 450–630 nm. Moreover, a complementary absorption increases the overlap of acceptor emission and donor absorption and enhances the energy transfer from the acceptor to the donor (Förster Resonant Energy Transfer, FRET). In our case, this would leave the Δ_HOMO_ offset of the blend unimportant and could have a positive effect on the charge generation efficiency as discussed in ref. 13. A possible design strategy to increase the bandgap of the PMI-FF-PMI molecule is to replace the perylene monoimide unit with a smaller conjugated molecule (*e.g.* naphthalene monoimide). Alternatively, it has been shown that increasing the lateral extension of the perylene core can lead to a stronger UV absorption.^47,48^ In addition, the usage of ester rather than imide end groups might present another possibility to increase the bandgap of perylene-based acceptors.^49,50^

## 4 Summary & conclusion

In summary, the non-fullerene acceptor PMI-FF-PMI based on two perylene monoimide units bridged *via* a dihydroindeno[1,2-*b*]fluorene linker exhibits excellent absorption and emission properties in the visible regime and shows good solubility and film formation properties. The PMI-FF-PMI acceptor was used in combination with the commercially available, high-performance donor polymer D18 to fabricate organic BHJ solar cells. Electrochemical measurements (EVS & CV) were used to determine the energy levels of donor and acceptor molecules. HOMO energy levels of −5.62 eV and −5.80 eV and LUMO_opt_ energy levels of −3.58 eV and −3.74 eV were obtained for D18 and PMI-FF-PMI, respectively. This material combination thus represents a wide band gap D/A blend with small Δ_HOMO_ and Δ_LUMO_ offsets. *J*–*V*-response measurements reveal that D18:PMI-FF-PMI solar cells are characterized by an extremely high *V*_OC_ of 1.41 V, which to the best of our knowledge is the highest *V*_OC_ value reported for solution-processed, single-junction organic solar cells to date. A maximum EQE_PV_ of 52% indicates moderately efficient charge generation in this D/A blend. PLQY quenching and PL lifetime measurements were performed to investigate the charge transfer efficiency of D18:PMI-FF-PMI. Both experiments suggest a lower driving force for charge transfer compared to highly efficient D18:Y6 or D18:PC_71_BM blends. The low driving force was identified as the main factor restricting the maximum EQE_PV_ of D18:PMI-FF-PMI solar cells. Both, highly sensitive EQE_PV_ and EL measurements did not show any additional CT absorption or emission features. Thus, the presented CT state analysis could not be used to determine an exact value for the CT state energy. Nevertheless, the experimental results indicate a small offset between the LE and CT state. Finally, the photovoltaic parameters and the open-circuit voltage losses of D18:PMI-FF-PMI solar cells were analyzed and compared to high-performance D18:Y6 and D18:PC_71_BM devices, representing state-of-the-art fullerene and non-fullerene based OSCs. Despite its high *V*_OC_, the D18:PMI-FF-PMI suffers from significantly higher Δ*V*^non-rad^_OC_ losses (0.25 V) compared to the solar cell based on D18:Y6 (0.20 V). It was found that the ELQY measured for D18:PMI-FF-PMI OSCs is significantly higher than the ELQY values derived from the non-radiative voltage losses.

To conclude, the effective bandgap of about 2.02 eV and the extremely high *V*_OC_ make D18:PMI-FF-PMI solar cells ideal candidates for the application as a wide-bandgap sub-cell in all-organic triple-junction devices. Considering the large potential to optimize the CT driving force of the D18:PMI-FF-PMI blend in combination with the great possibilities to fine-tune optical and electronic properties of the perylene π-system, we hope that our work can help to accelerate the development of future wide-bandgap NFAs. In addition, our experimental results reveal that in exceptional cases the measured ELQY of a solar cell device cannot be used to derive its non-radiative voltage loss. ELQY measurements of D18:PMI-FF-PMI solar cells show that ELQY_meas_ might overestimate the actual radiative efficiency of the solar cell by more than a magnitude. The direct injection of electrons in the LUMO of the donor polymer or holes in the HOMO of the acceptor molecule are presented as possible explanations for the enhanced ELQY values. Regardless, the large discrepancy between ELQY_meas_ and ELQY_calc_ values observed in D18:PMI-FF-PMI solar cells strongly suggests that solely relying on the measured ELQY can lead to severe misinterpretation of the observed Δ*V*^non-rad^_OC_ loss. Therefore, our results should encourage OPV researchers to always validate the ELQY_meas_ values with the ELQY_calc_ values derived from a voltage loss analysis.

## 5 Methods

### 5.1 Materials & device preparation

D18 and Y6 were purchased from 1-materials, while PC_71_BM was purchased from Solenne BV. Before the solar cell fabrication, pre-patterned ITO glass was first wiped with toluene, followed by subsequent ultrasonication in Hellmanex (2% v/v solution in deionized water, 50 °C), 2× in deionized water, acetone and isopropanol. Each sonication step was performed for 15 min. Following the cleaning process, the substrates were blow-dried with N_2_ followed by an O_2_ plasma treatment for 5 min at 100 W. Solar cells in the standard configuration (ITO/PEDOT:PSS/absorber layer/Ca/Al) were prepared spin coating a 0.45 μm filtered PEDOT:PSS solution (Clevios Al4083) onto the clean substrates. A recipe with 3000 rpm for 45 s was used to obtain films thicknesses of 30–40 nm. The PEDOT:PSS films were annealed at 150 °C for 10 min to remove residual water and were then transported into a nitrogen-filled glove box where the active layer was spin-coated. The active layer solution of D18:PMI-FF-PMI was prepared in chlorobenzene with a D/A weight ratio of 1 : 1 and a total concentration of 13.3 mg mL^−1^. The active layer solution was prepared from master solutions of D18 (10 mg mL^−1^ in chlorobenzene) and PMI-FF-PMI (15 mg mL^−1^ in chlorobenzene). Before use the D18 and D18:PMI-FF-PMI solutions were stirred at 90 °C for 30 min in order to fully dissolve the polymer. The active layer was spin-coated at 60 °C with a two-step recipe of 1500 rpm for 2 s and 4000 rpm for 20 s. The spin-coating recipe resulted in film thicknesses between 70–90 nm. The substrates were transferred to a thermal evaporator under a dry nitrogen atmosphere, where a 10 nm Ca and a 100 nm Al layer were deposited at a pressure <10^−6^ mbar using a shadow mask. The active area of the cells was around 0.1 cm^2^. The exact area of each solar cell was determined with an optical microscope. All cells were encapsulated in the glovebox using a two-component epoxy sealant. The device preparation of D18:PMI-FF-PMI cells in the inverted device structure is summarized in Note S7, ESI.† The detailed fabrication of the D18:Y6 and D18:PC_71_BM solar cells is reported elsewhere.^9^

### 5.2 Solar cell characterization

*J*–*V*-measurements of the solar cell devices were performed with a LOT-QD solar simulator (LS0821) and a Keithley 2401 Source-Meter unit. The intensity was calibrated using a reference Si-diode and set to 100 mW cm^−2^ (=AM1.5G). A custom-built LabVIEW software was used to record the current–voltage curves under AM1.5G illumination and in the dark.

### 5.3 EQE_PV_

The custom-built EQE_PV_ setup consists of a xenon lamp, a monochromator (Oriel Cornerstone), a Jaissle 1002 potentiostat, and a lock-in amplifier (SR830, Stanford Research Systems). The mechanically chopped light from the xenon lamp was coupled into the monochromator and a set of long-pass filters was used to guarantee monochromatic illumination of the device. The Jaissle potentiostat was used as a current to voltage converter with variable gain ranging from 10 to 10^8^ VA^−1^. The combination of phase-sensitive lock-in detection and variable preamplification enables highly sensitive EQE_PV_ measurements. Typically, the EQE_PV_ measurements presented in this work consist of two separate measurements. In addition to the standard EQE_PV_ measurement, a second measurement with a 610 nm long-pass filter and increased preamplification in the range from 630–800 nm is performed to analyze the behavior below the band edge. The xenon lamp spectrum was corrected using a calibrated silicon diode (Hamamatsu S2281) as a reference.

To estimate the IQE_PV_ of the solar cells, first, the EQE_PV_ was measured under an angle of 13°, followed by a measurement of the reflected light intensity using the calibrated Si-diode. The reflected light intensity is used to determine the spectral reflectance of the solar cell. Assuming a perfectly reflecting back-electrode and neglecting parasitic absorption and light scattering allows to derive a lower estimate for the IQE_PV_ of the solar cell.

### 5.4 Optical characterization

A Lambda 1050 double-beam UV-vis-NIR spectrometer from PerkinElmer was used to determine the optical transmission *T* and absorbance *A* of the organic thin films on glass. A specular reflectance module to measure the reflectance *R* under a 6° incidence angle was used for the reflectance measurements to calculate the absorption coefficient using 
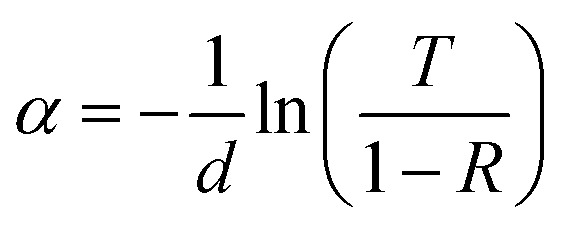
, where *d* is the film thickness measured with a DekTakXT stylus profilometer (Bruker). Excitation and emission spectra of donor and acceptor thin films were measured with a PTI QuantaMaster 40 fluorescence spectrometer. An Andor Shamrock SR-303i monochromator and an Andor Peltier-cooled iDus Si-CCD (420-OE) were used for photoluminescence and electroluminescence measurements. The measurement setup was calibrated with a tungsten-halogen source (Ocean Optics HL-2000) to determine the overall spectral response of the monochromator/detector system. For PL measurements the devices were excited with a solid-state laser (Coherent OBIS 488 nm LX 150 mW) with a wavelength of 488 nm. The optical output power of the laser was adjusted to obtain similar currents as measured under AM1.5G illumination. A 550 nm long-pass filter in front of the monochromator was used to successfully suppress the 488 nm laser light and allowed the acquisition of a PL spectrum without the influence of the excitation light. For EL measurements a Keithley 2401 Source Meter Unit (SMU) was used to apply different potentials to the solar cells. The injection currents were set to match the observed photocurrents under AM1.5G illumination. The absolute photoluminescence quantum yield was measured using a Hamamatsu C9920-03 spectrometer with an integrating sphere.

### 5.5 Electrochemistry

Electrochemical voltage spectroscopy (EVS) and cyclic voltammetry (CV) measurements were performed using a Jaissle Potentiostat–Galvanostat IMP 88 PC-100. A three-electrode setup with an Ag/AgCl wire as quasi-reference, a Pt plate as counter, and a Pt plate covered with the organic material as working electrode was used. The organic materials were drop-cast onto the Pt-electrode from chloroform solution under N_2_ atmosphere. All EVS measurements were performed in a nitrogen-filled glovebox using 0.1 M tetrabutylammonium hexafluorophosphate (TBAPF6) in acetonitrile (MeCN) as the electrolyte solution. During the EVS measurements, the applied potential is stepwise increased or decreased by 10 mV. The current at each potential step is measured for 20 s and integrated to obtain the amount of charge (Δ*Q*) passing through the system for each voltage step. In the absence of an electrochemical reaction, no net current is observed, leading to a constant baseline of Δ*Q*. The oxidation and reduction onsets of the materials were determined either at the position where Δ*Q* starts to deviate from the baseline (lower limit) or at the crossing point of two tangents drawn through the baseline and the slope of the reaction peak (upper limit). Measurements in the reductive and oxidative regime were performed on separate substrates to avoid any hysteresis. Every measurement was externally calibrated by measuring the half-wave potential of a ferrocene/ferrocenium (Fc/Fc^+^) redox couple. The measured Fc/Fc^+^ half-wave potential was used to correct the reference electrode and plot the measured data referenced to the normal hydrogen electrode (NHE). An oxidation potential for Fc/Fc^+^*vs.* normal hydrogen electrode (NHE) of 400 mV was used.^51^ The Fermi level of NHE *vs.* vacuum was taken as −4.75 eV.^52^

### 5.6 ELQY

Measurements to estimate the ELQY were performed using a calibrated, large area Si-photodiode (Hamamatsu S2281). The organic solar cell (∼0.1 cm^2^) was positioned in the center and directly in front of the large area Si photodiode (1 cm^2^). A Keithley 2401 SMU was used to operate the solar cell as a LED, while another Keithley 2401 SMU was used to measure the photocurrent of the Si-photodiode. A detailed description of the used analysis procedure to determine the ELQY of the solar cells is provided elsewhere.^9^

### 5.7 Time correlated single photon counting

Time-resolved measurements were performed in a closed-cycle helium cryostat (Oxford OptistatDry) using a time-correlated single-photon counting setup consisting of a DeltaNu DNS-300 monochromator, a Becker & Hickl SPC 150 TCSPC module, and a PMC-100-1 cooled photomultiplier. A supercontinuum laser (NKT Photonics SuperK FIANIUM FIU-15) equipped with a pulse picker and a wavelength selection unit (Photonetc LLTF CONTRAST VIS) was used as an excitation light source. The presented measurements were performed at room temperature and ambient conditions. The decay time, where the intensity is reduced to 1/*e* of its initial value, was used to estimate the PL lifetime.

## Conflicts of interest

There are no conflicts to declare.

## Supplementary Material

TA-010-D1TA09752K-s001
