# Supplementary material for: Wide-bandgap organic solar cells with a novel perylene-based non-fullerene acceptor enabling open-circuit voltages beyond 1.4 V
Source: J Mater Chem A Mater. 2021 Dec 15;10(6):2888–906. doi: 10.1039/d1ta09752k (PMC8823902; doi:10.1039/d1ta09752k)
Supplement: TA-010-D1TA09752K-s001 [file TA-010-D1TA09752K-s001.pdf]

Electronic Supplementary Material (ESI) for Journal of Materials Chemistry A.
This journal is © The Royal Society of Chemistry 2021

Wide-bandgap organic solar cells with a novel perylene-based non-fullerene acceptor enabling open-circuit voltages beyond 1.4 V.

Jakob Hofinger[1], Stefan Weber[2], Felix Mayr[3], Anna Jodlbauer[2], Matiss Reinfelds[2], Thomas Rath[2], Gregor Trimmel[2], Markus C. Scharber[1]**

[1] Linz Institute of Organic Solar Cells (LIOS), Institute of Physical Chemistry, Johannes Kepler University Linz, Altenbergerstrasse 69, 4040 Linz, Austria

[2] Institute for Chemistry and Technology of Materials, NAWI Graz, Graz University of Technology, Stremayrgasse 9, 8010 Graz, Austria

[3] Institute of Applied Physics, Johannes Kepler University Linz, Altenbergerstrasse 69, 4040 Linz, Austria

Supporting Information:

Synthesis of PMI-FF-PMI

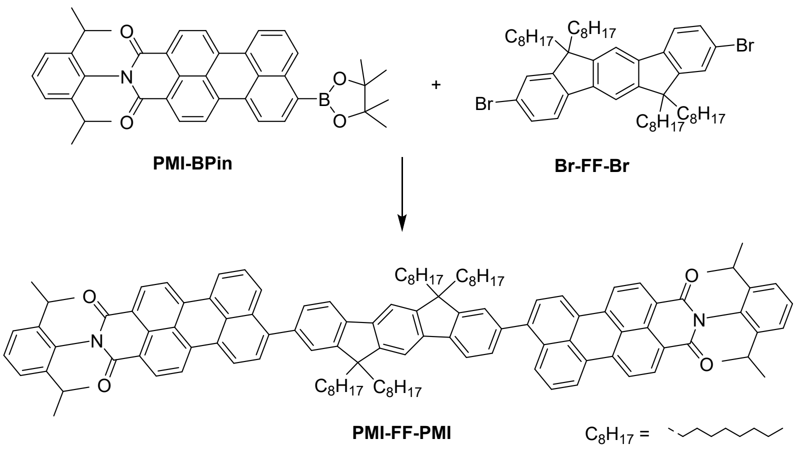

Figure S1: Synthesis scheme of PMI-FF-PMI.

Note S1:

In a Schlenk tube, operated under nitrogen, 500 mg (0.823 mmol, 2.1 equiv.) of 8-(4,4,5,5-tetramethyl-1,3,2-dioxaborolan-2-yl)-*N*-(2,6-diisopropylphenyl)-perylene-3,4-dicarboximide (**PMI-BPin**) and 337 mg (0.392 mmol, 1.0 equiv.) of 2,8-dibromo-6,6,12,12-tetraoctyl-6,12-dihydroindeno[1,2-b]fluorene (**Br-FF-Br**, CAS Number 264281-45-0) were dissolved in 50 mL toluene followed by the addition of 1M K_2CO_3 (5 ml) and 1 drop of Aliquat 336. Afterwards, $Pd(PPh_3)_4$ (0.039 mmol, 0.1 equiv.) was added and the reaction mixture was heated at 100 °C for 24 h. Upon completion, the reaction mixture was extracted with H_2O and dried over Na_2SO_4 followed by evaporation of the solvent under reduced pressure. The residue was purified by column chromatography (eluent: CH_2Cl_2/pentane – 10/1) and further recrystallized using CH_2Cl_2/hexane to yield **PMI-FF-PMI** as a violet solid. Yield: 228 mg (35%). R_f = 0.39 – 0.52 (CH_2Cl_2)

^{1}H NMR (500 MHz, $CDCl_3$): δ = 8.71 (dd, *J* = 7.8 Hz, 4H), 8.60 (d, *J* = 8.0 Hz, 2H), 8.58-8.52 (m, 6H), 8.11 (d, *J* = 8.5 Hz, 2H) 7.97 (d, *J* = 7.7 Hz, 2H) 7.79 (s, 2H), 7.73 (d, *J* = 7.7 Hz, 2H), 7.62 (t, *J* = 7.8 Hz 2H), 7.58 (dd, *J* = 7.7, 1.1 Hz, 2H), 7.56 (s, 2H), 7.49 (t, *J* = 7.8 Hz, 2H) 7.35 (d, *J* = 7.8 Hz, 4H), 2.79 (hept, *J* = 6.9 Hz, 4H), 2.18-2.09 (m, 8H), 1.24-1.10 (m, 64H), 0.90-0.78 (m, 20H) ppm. ^{13}C NMR (75 MHz, $CDCl_3$): δ = 164.2, 151.7, 150.9, 145.9, 144.3, 141.5, 140.6, 138.5, 138.0, 137.8, 133.1, 132.3, 132.3, 131.3, 130.8, 129.7, 129.6, 129.0, 128.7, 128.6, 128.5, 127.2, 127.1, 124.9, 124.2, 124.1, 123.8, 121.2, 121.0, 120.5, 120.2, 119.8, 114.5, 55.2, 40.8, 32.0, 30.2, 29.5, 29.4, 29.3, 24.2, 22.8, 14.3 ppm. HRMS (MALDI-TOF) calc. for $C_{120}H_{128}N_2O_4H$ 1662.9987, found 1662.9929.

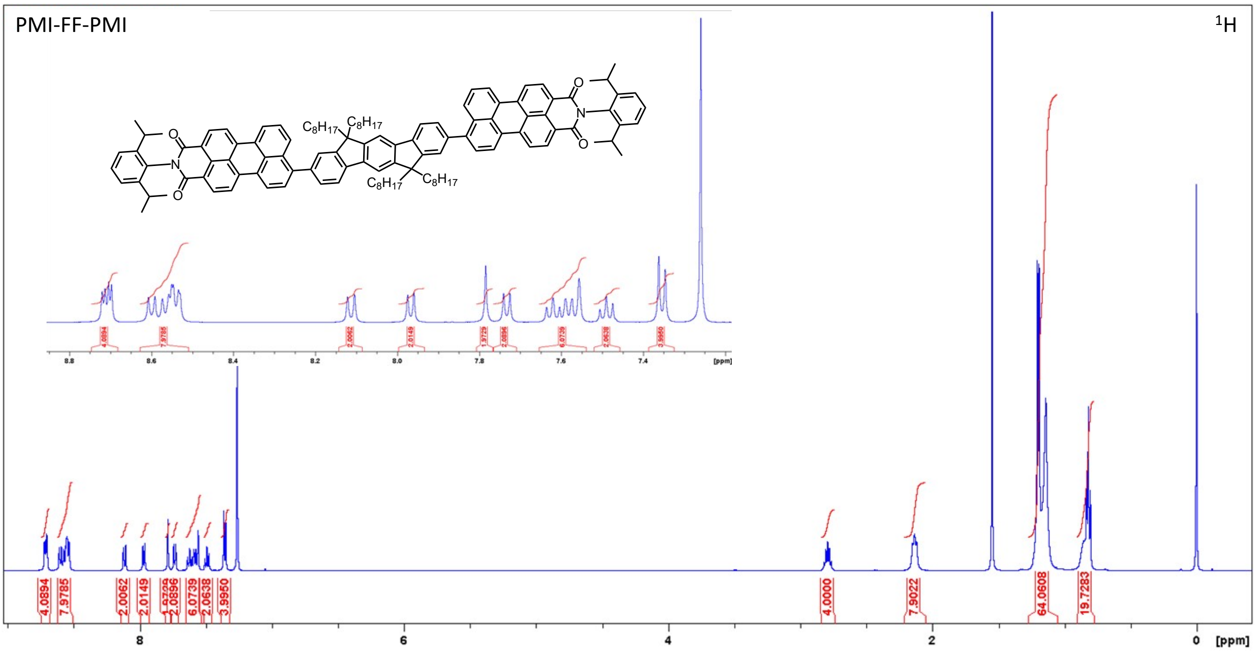

Figure S2: ^{1}H NMR (500 MHz, $CDCl_3$) spectrum of **PMI-FF-PMI** with an inset of the aromatic region, referenced to TMS.

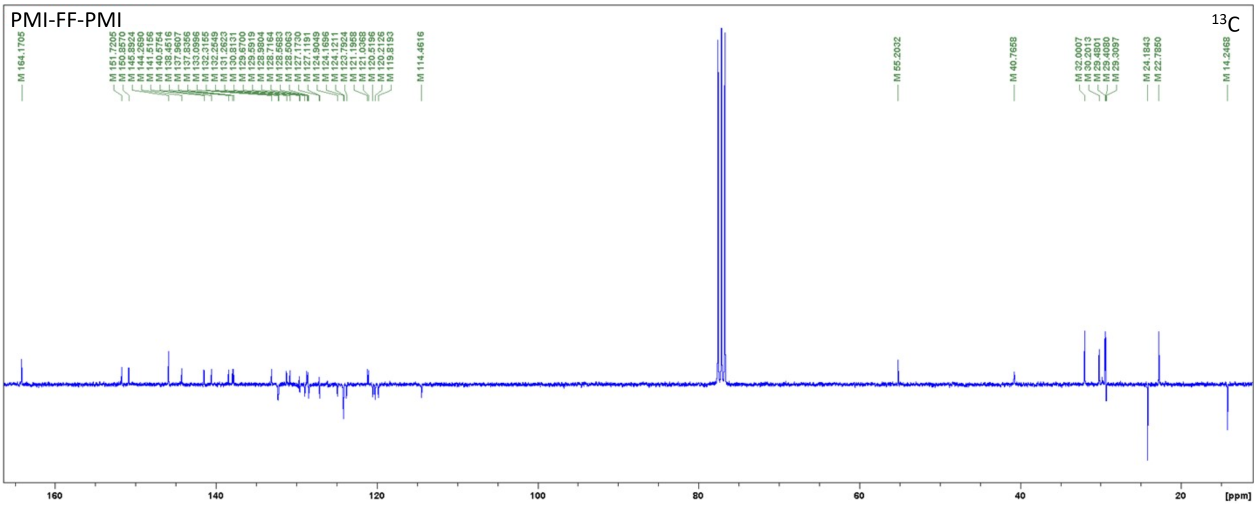

Figure S3: ^{13}C APT NMR (75 MHz, $CDCl_3$) spectrum of **PMI-FF-PMI**, referenced to $CDCl_3$.

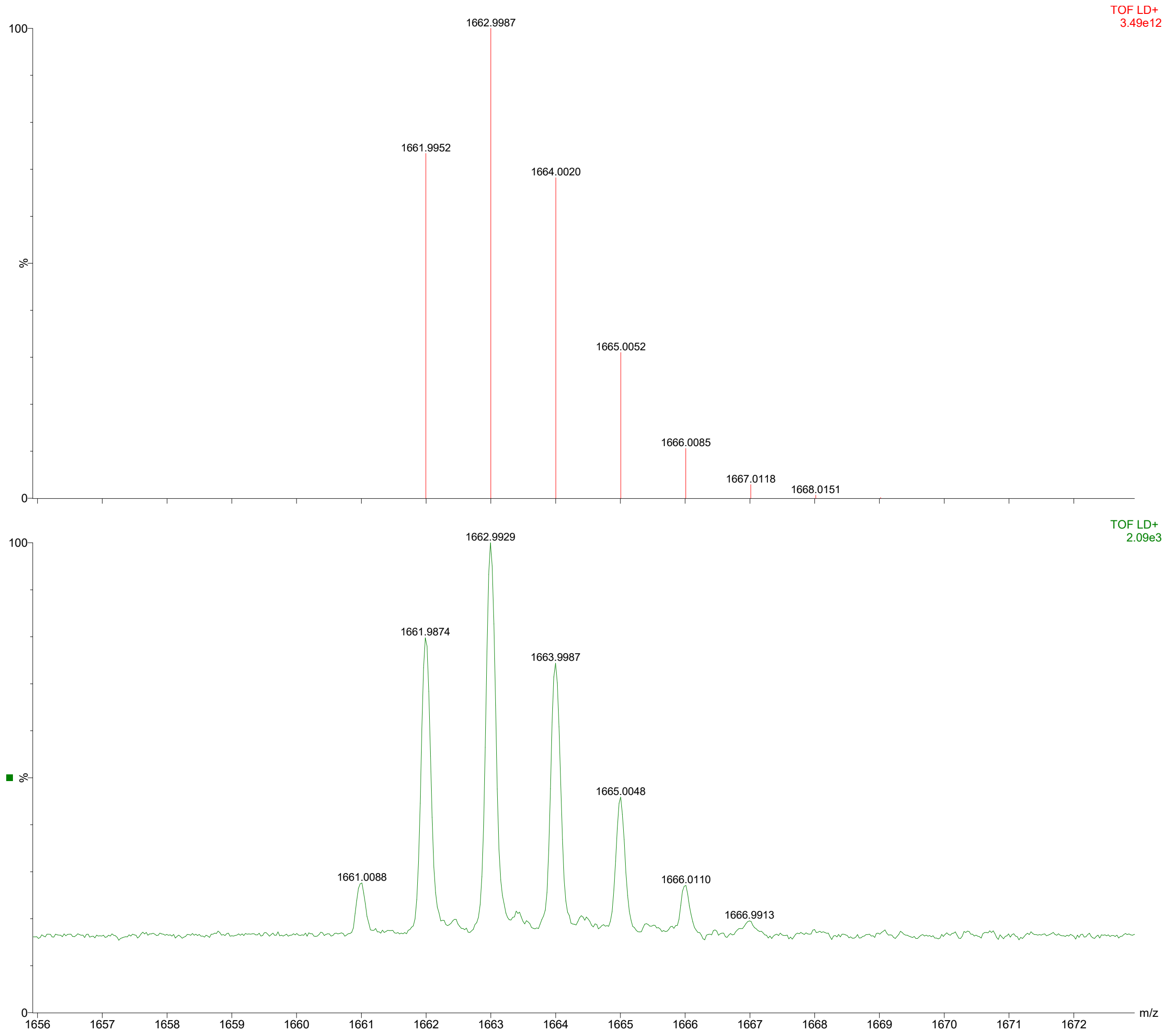

Figure S4: HRMS (MALDI-TOF, Dithranol matrix) of **PMI-FF-PMI**, upper – simulated, lower - found

Additional Optical Characterizations

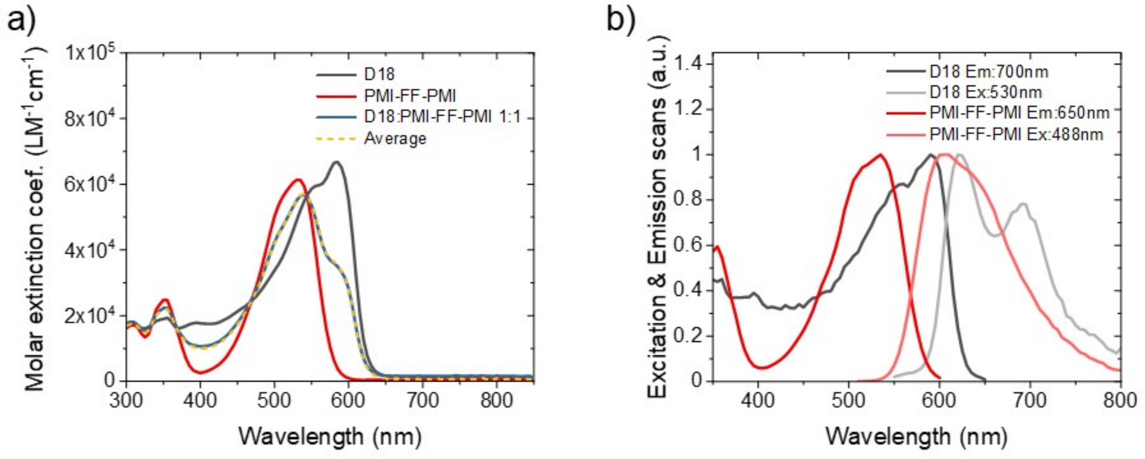

Figure S5: Optical characterization of D18 and PMI-FF-PMI solutions dissolved in chlorobenzene. a) Molar extinction coefficient obtained from absorbance measurements. The average spectrum of the two pristine materials (yellow dashed line) coincides with the measured 1:1 mix of D18 and PMI-FF-PMI. b) Excitation and emission scans of D18 and PMI-FF-PMI solutions. Excitation wavelengths during emission, and detection wavelength during excitation scans are presented in the legend.

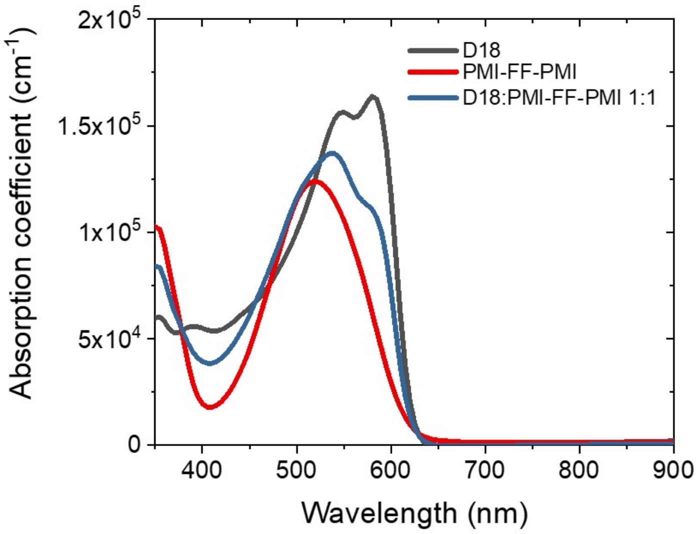

Figure S6: Absorption coefficients of D18, PMI-FF-PMI, and D18:PMI-FF-PMI (1:1) calculated from transmission and reflection measurements of thin films on glass substrates.

EVS & CV Measurements

Note S2:

Note S2.1: Evaluation procedure

An exact determination of the energy levels of donor and acceptor is of key importance for OPV performance analysis and future material design. Fine-tuning of the energy levels is necessary to find the optimal balance between small $\Delta V_{OC}^{non\text{-}rad}$ losses (low $\Delta E_{LE\text{-}CT}$ offset) and sufficient driving force for charge generation (large $\Delta E_{LE\text{-}CT}$ offset). Contradicting reports for HOMO and LUMO levels of organic materials suggest that commonly used techniques such as ultraviolet electron spectroscopy (UPS) or cyclic voltammetry (CV) suffer from large measurement and evaluation errors.[1,2] Depending on the used method, HOMO and LUMO levels may vary significantly, often leading to deviations in the magnitude of several tenths of eV. The difficulty in defining the reduction and oxidation onsets from electrochemical measurements is considered as the main source for the evaluation errors. A so-called tangent evaluation method is commonly used, where a tangent is fitted to the slope of the CV peak and the baseline, respectively. The intersection point is considered as the onset of the electrochemical reaction. Another possibility often used to evaluate EVS data, is to define the first deviation from the baseline as the reduction/oxidation onset. In **Figure 2**c, the results from the "first deviation method" were used to plot the box diagram. The whiskers represent the values obtained from the "tangent evaluation method" and should be considered as a maximum evaluation error, which can still be justified by the measurement data. A detailed illustration of the different evaluation methods is presented in *Note S2.2*. **Figure 2**c shows that regardless of the evaluation method the HOMO level of donor and acceptor can be determined with good accuracy. The determination of the LUMO levels on the other hand shows a strong variation between the evaluation methods. Especially the LUMO of the polymer is hard to identify, which is further discussed in *Note S2.2*. Reliable measurements of the HOMO energy levels and difficulties to determine the LUMO energy levels suggest estimating the LUMO level by adding the respective optical bandgap (E_{opt}) to the HOMO energy levels of donor and acceptor ($LUMO_{opt}$).

Note S2.2: EVS vs. CV measurements

Typically, a tangent method, as described in *Note S2.1*, is used to determine the reduction and oxidation onsets from CV measurements. As it can be seen from the CV oxidation peaks in

Figure S7a, determining the tangent is often ambiguous and leaves great scope for interpretation. To reduce evaluation errors, EVS measurements were used as an alternative to estimate the HOMO and LUMO levels of OPV materials. In EVS, the onset of reduction or oxidation is simply defined as the first deviation from the baseline, which significantly reduces the evaluation uncertainty. Furthermore, EVS is used to measure specifically the start of the electrochemical reaction and is thus ideally suited to investigate reduction and oxidation onsets. As shown in **Figure S7**a, the reaction onsets measured in EVS can be associated with the oxidation and reduction peaks in CV experiments. In the following, the CV and EVS measurements were evaluated with both methods. The results from the 1st deviation method should be regarded as a lower limit for the energy levels of donor and acceptor (box), while the tangent method should be considered as an upper limit (whiskers). **Figure S7**b shows the evaluation of the EVS data for D18 and PMI-FF-PMI, respectively. The analysis shows that the HOMO levels of both, donor and acceptor, can be determined with good accuracy regardless of the evaluation method. On the contrary, the determination of the LUMO level of D18 is difficult due to a delayed reduction onset. The delayed onset leads to a large discrepancy between the values obtained from the 1st deviation and the tangent method. To a lesser extent, the same is true for the PMI-FF-PMI acceptor. To avoid this problem the LUMO levels of both materials were calculated by adding the optical bandgaps E_{opt} to the respective HOMO levels. $LUMO_{opt}$ is represented by the yellow bars in **Figure S7**b&c. It is worth noting that the delayed reduction onset for the D18 polymer is not displayed in the CV data and leads to an electrochemical bandgap which is approximately 0.5 eV larger than the optical bandgap. A similar delayed reductive behavior has been observed for all the investigated donor polymers (PTB7, PTB7-Th, PBDB-T, PM6, PTQ11, PTZ1; not shown here). Our results suggest that CV measurements largely overestimate the LUMO levels of state-of-the-art donor polymers. **Figure S7**c gives a summary of the derived energy levels for D18 and PMI-FF-PMI using both methods and evaluation techniques. A comparison of the HOMO levels suggests that for both donor and acceptor the EVS data exhibits less dependency on the evaluation method and validates the usage of the EVS method over the CV method.

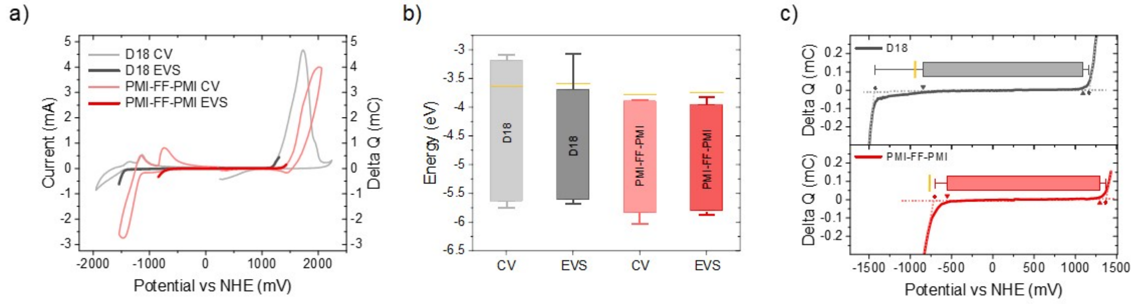

Figure S7: Electrochemical characterization of D18 and PMI-FF-PMI films. a) Comparison of EVS and CV measurements of D18 and PMI-FF-PMI. b) Summary of the derived HOMO and LUMO levels from CV and EVS measurements of D18 and PMI-FF-PMI. c) Graphical illustration of the two discussed evaluation methods. The values obtained from the 1st deviation and tangent method are indicated by the red and black triangle and diamond symbols, respectively. The LUMO level, determined from the HOMO level and the optical bandgap, is highlighted in yellow.

D18:PMI-FF-PMI solar cells fabricated in the inverted structure

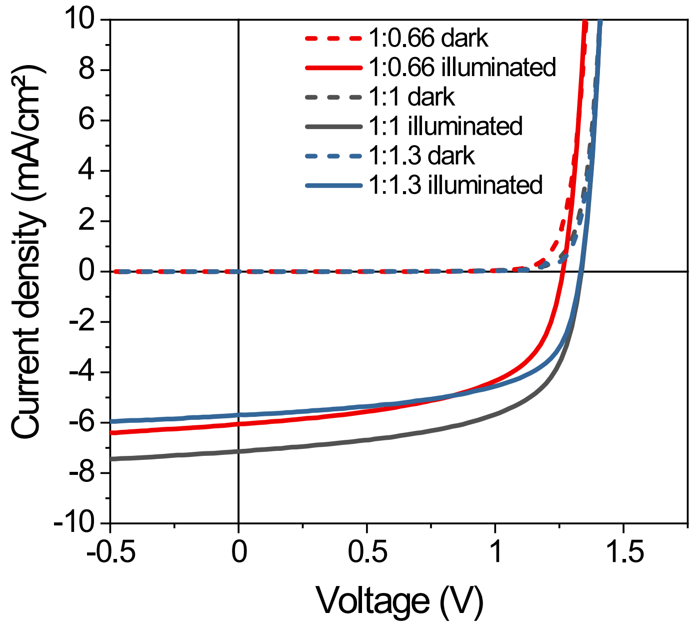

Figure S8: *J-V* characteristics measured in the dark and under 100 mW/cm² AM1.5G illumination of typical D18:PMI-FF-PMI based solar cells prepared in the inverted device architecture and with different D/A ratios (see also **Table S1**).

Table S1: Characteristic device parameters of D18:PMI-FF-PMI based solar cells prepared in inverted architecture (glass/ITO/ZnO/D18:PMI-FF-PMI/MoO_3/Ag). The D/A weight ratio was varied from 1:0.66 to 1:1.33.

D:A ratio		V_{OC} (V)	J_{SC} (mA cm^{-2})	FF (%)	PCE (%)
1:0.66	best cell	1.26	6.0	57.1	4.32
	average	1.24 ± 0.01	5.7 ± 0.2	55.8 ± 1.1	3.92 ± 0.21
1:1	best cell	1.34	7.1	60.1	5.69
	average	1.35 ± 0.01	6.4 ± 0.5	59.8 ± 2.2	5.13 ± 0.32
1:1.3	best cell	1.34	5.7	60.9	4.65
	average	1.32 ± 0.03	5.3 ± 0.3	56.8 ± 6.0	4.00 ± 0.51

Light dependent *I-V* measurements

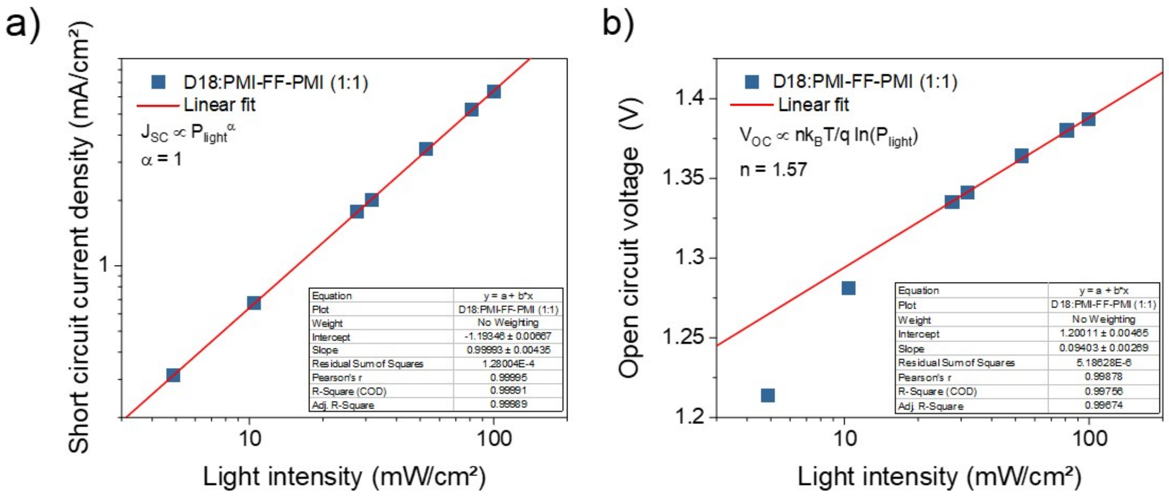

Figure S9: Intensity-dependent J_{SC} and V_{OC} measurements.

Mobilities

Note S3:

The OFET devices were fabricated in a bottom gate, top contact geometry. The aluminum bottom gate electrode was thermally evaporated onto a glass substrate. A 32 nm aluminum oxide layer (dielectric layer) was obtained by electrochemical anodization. Benzocyclobutene (BCB) was used as a dielectric passivation layer. The PMI-FF-PMI acceptor was spin coated (33rps for 30s) from a 9 mg/mL chloroform solution resulting in typical thicknesses of around 75 nm. Finally, aluminum source and drain top contacts were thermally evaporated resulting in transistors with a channel width *W* of 2000 µm and a channel length *L* of 65 µm. The device structure and the detailed fabrication process of the electrodes and dielectric layers is described elsewhere.[3]

In the saturation regime the drain current I_{drain} can be described with

$$I_{drain} = \frac{WC_{ox}}{2L} \cdot \mu_e^{sat} (V_{gate} - V_{Th})^2, \qquad \textbf{(S1)}$$

where C_{ox} is the capacitance per unit area, μ_e^{sat} is the electron saturation mobility, V_{gate} is the gate and V_{Th} is the threshold voltage. Rearranging equation S1 allows to calculate the saturation mobility from the slope of the (V_{gate}-V_{Th}) *vs.* $\sqrt{I_{drain}}$ plot:

$$\mu_e^{sat} = \frac{2L}{WC_{ox}} \left(\frac{\partial \sqrt{I_{drain}}}{\partial (V_{gate} - V_{Th})} \right)^2 \qquad \textbf{(S2)}$$

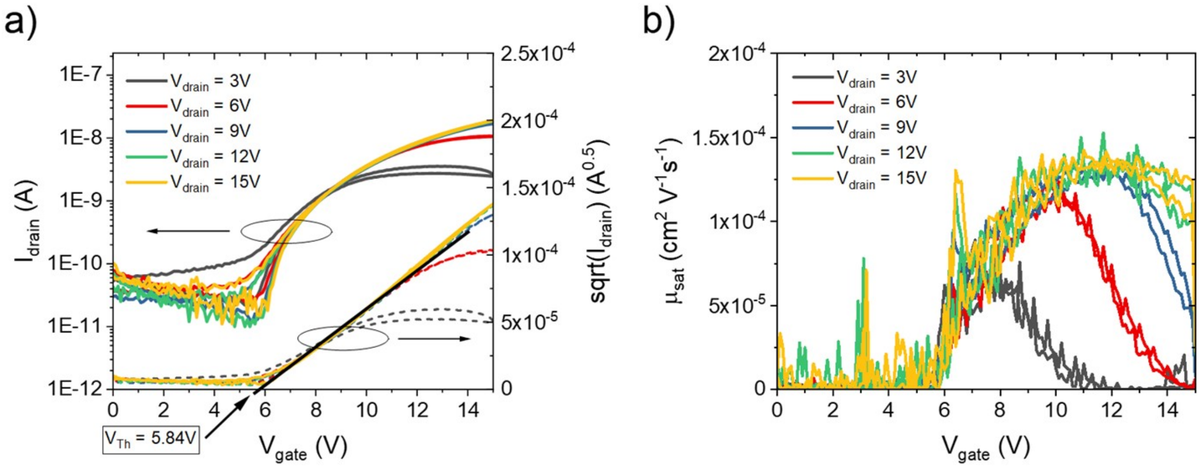

Figure S 10: OFET transfer characteristic and saturation mobility. a) Transfer characteristic of a PMI-FF-PMI OFET. The devices were measured with an Agilent B1500A semiconductor device parameter analyzer. The OFET transfer curves were measured by sweeping the gate voltage from 0 to 15 V and back to 0 V in 100 mV steps. After each sweep the drain voltage was increased by 3 V until a final drain voltage of 15 V was reached. The solid curves indicate the drain current on a logarithmic scale (left y-axis), while the dashed curves represent the square root of the drain current on a linear scale (right y-axis). The straight black line indicates the linear fit of $\sqrt{I_{drain}}$. The intersection of the black line with the x-axis allows to read out the

values of the threshold voltage V_{Th}. b) The measurements presented in a) were used to derive the saturation mobility curves following equation S2.

Bias PL reduction of D18:Y6

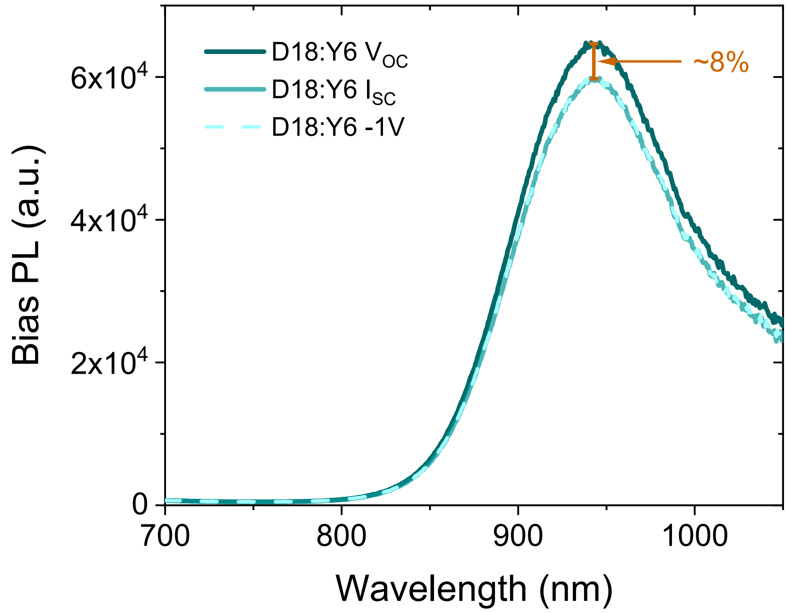

Figure S11: Bias dependent Photoluminescence. PL of a high efficient D18:Y6 solar cell with D/A ratio 1:1.6. The detailed device fabrication and characterization of the solar cells are reported in Reference 4. Measured PL signal decreases about 8% upon changing bias conditions from V_{OC} to I_{SC}. A further increase to reverse bias conditions (-1 V) does not alter the PL signal.

D18:PMI-FF-PMI optimization

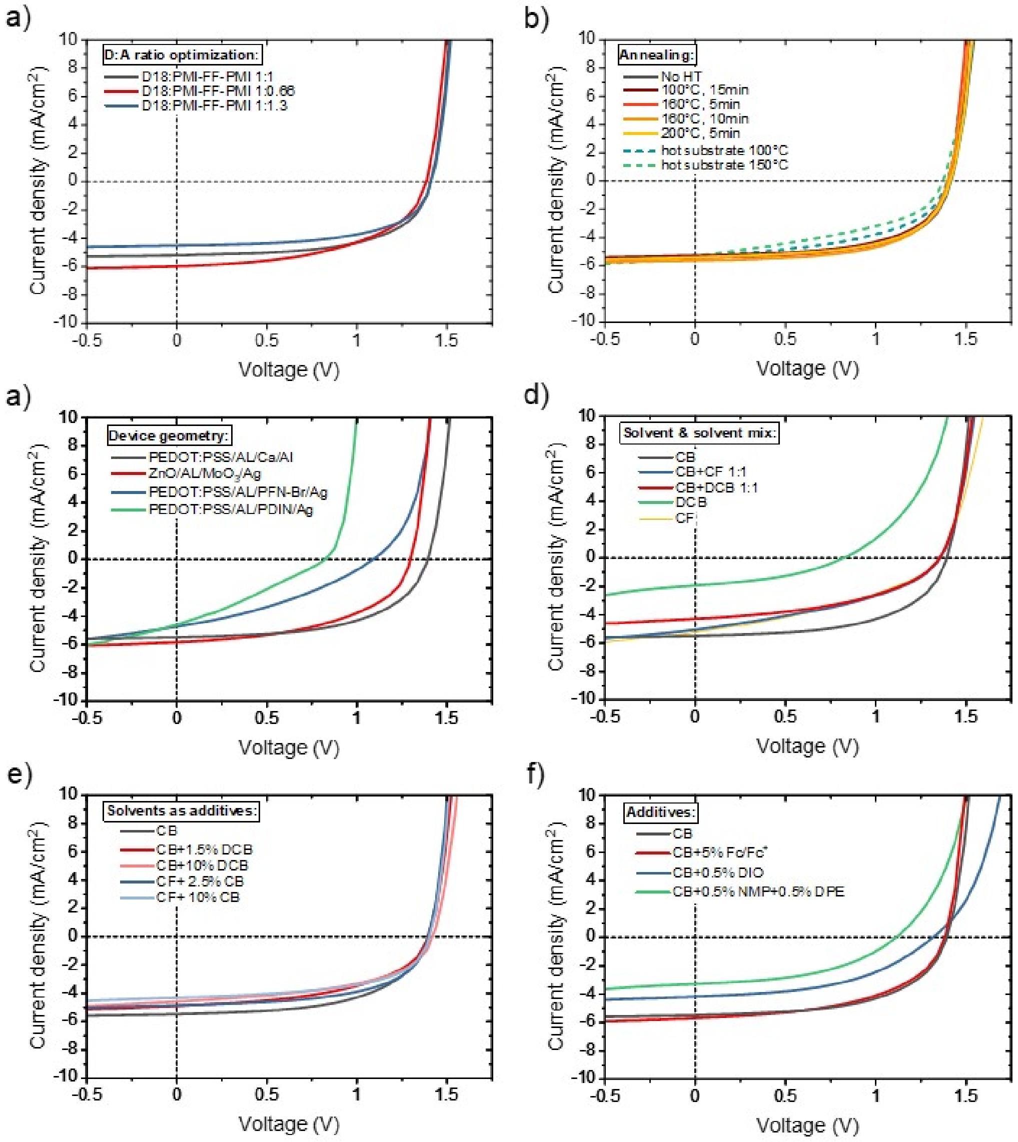

Figure S12: D18:PMI-FF-PMI solar cell device optimizations. a) *J*-*V*-curves of solar cells with varying D/A ratios. b) *J*-*V*-curves of D18:PMI-FF-PMI 1:1 solar cells upon changing the annealing temperature and time. The dashed lines represent *J*-*V*-curves where the substrates have been heated prior to the spin-coating process. c) *J*-*V*-curves of D18:PMI-FF-PMI solar cells with different device geometry. d) *J*-*V*-curves of D18:PMI-FF-PMI 1:1 solar cells fabricated using different organic solvents and solvent mixtures including chlorobenzene (CB), chloroform (CF), and dichlorobenzene (DCB). e) *J*-*V*-curves of D18:PMI-FF-PMI 1:1 solar cells using CB and CF as solvents and adding DCB and CB as respective additives. f) *J*-*V*-curves of D18:PMI-FF-PMI 1:1 solar cells using CB as a solvent and adding solvent additives like ferrocene (Fc/Fc^+), diiodooctane (DIO) and N-methyl pyrrolidone (NMP), and diphenyl ether (DPE). The presented *J*-*V*-curves are averaged over at least 6 solar cells.

As shown in **Figure S12**a, varying the D/A weight ratio of D18:PMI-FF-PMI solar cells only slightly affects the solar cell performance. D/A blends with a ratio of 1:0.6 exhibit the highest J_{SC} values but suffer from a reduced V_{OC} and FF. On the contrary, if the ratio is changed to 1:1.3 the solar cell FF slightly increases compared to the 1:1 blend, but the J_{SC} is continuously reduced. Overall, the optimum performance was found for solar cells with a balanced D/A ratio of 1:1. No further improvements were obtained upon annealing of the D/A blend prior to the evaporation of the top contact. Extraordinary thermal stability of D18:PMI-FF-PMI solar cells was observed showing no significant change up to annealing temperatures of 200 °C. Pre-heating of the substrates prior to the spin coating process resulted in D/A films with an increased thickness and is the reason for the slightly reduced FFs in those solar cells. As seen in **Figure S12**c, the device structure with PEDOT:PSS as hole transport layer (HTL) and Ca as electron transport layer (ETL) resulted in solar cells with the best performance. Inverting the structure and using ZnO as ETL and MoO_3 as HTL slightly increased the current but led to reduced V_{OC} and FF values. Replacing Ca with ETLs like PDIN or PFN-Br significantly reduced the performance. Most probably the high-work function Ca layer is needed to form an ohmic contact with the wide-bandgap (high LUMO) acceptor PMI-FF-PMI. Various tries to use different solvents, solvent mixtures or solvent additives did not result in an improvement in either V_{OC}, J_{SC}, or FF as depicted in **Figure S12**d-f.

Calculation of radiative, non-radiative, and quenching rates

Note S4:

According to equation (6), the PLQY of the pristine D18 film can be used to express the ratio between k_{nr} and k_r as

$$\frac{k_{nr}}{k_r} = \frac{1 - PLQY_{pristine}}{PLQY_{pristine}} \quad \textbf{(S3)}$$

The denominator in equation (6) can be interpreted as the TCSPC lifetime of the pristine D18 film (see equation (S5)). Therefore, τ_{meas} of D18 can be used to calculate the absolute values of k_{nr} and k_r. With the knowledge of k_{nr} and k_r the quenching rate k_q can be determined from equation (S3) as follows:

$$k_q = \frac{k_r - k_r \cdot PLQY_{blend} - k_{nr} \cdot PLQY_{blend}}{PLQY_{blend}} \quad \textbf{(S4)}$$

Table **NoteS4_T1** summarizes the derived values for the D18:PMI-FF-PMI films with varying acceptor concentrations from 1 % to 50 %.

NoteS4_T1: Calculated radiative, non-radiative, and quenching rates of D18:PMI-FF-PMI films with varying acceptor concentrations. Equation (S5) was used to calculated τ_{calc}.

D/A ratio	*PLQY* (%)	k_{nr}/k_r (a.u.)	τ_{meas} (ns)	k_r^{calc} (s^{-1})	k_{nr}^{calc} (s^{-1})	k_q^{calc} (s^{-1})	τ_{calc} (ns)
D18 pristine	14.4	5.9	0.62	$2.32 \cdot 10^8$	$1.38 \cdot 10^9$	-	0.62
99:1	11.9	-	-	$2.32 \cdot 10^8$	$1.38 \cdot 10^9$	$3.4 \cdot 10^8$	0.51
9:1	6.6	-	-	$2.32 \cdot 10^8$	$1.38 \cdot 10^9$	$1.9 \cdot 10^9$	0.28
3:1	3.9	-	-	$2.32 \cdot 10^8$	$1.38 \cdot 10^9$	$4.3 \cdot 10^9$	0.17
1:1	3.7	-	-	$2.32 \cdot 10^8$	$1.38 \cdot 10^9$	$4.7 \cdot 10^9$	0.16

TCSPC lifetime of D18:PMI-FF-PMI blends

Note S5:

In a simple model, the denominator of equation (5) can be identified as the PL lifetime (τ) of a D/A blend,

$$\tau = \frac{1}{\sum_i k_i} = \frac{1}{k_r + k_{nr} + k_q} \quad \textbf{(S5)}$$

and allows the calculation of τ_{calc} from the rate constants derived as described in **Note S4** and presented in **Table 3**. A comparison of the measured and calculated lifetimes of the three 99:1 D/A films as shown in **NoteS5_T1** indicates that the measured lifetime is significantly larger than the calculated one for all the investigated blends. The large discrepancy between the measured and calculated lifetime suggests that equation (S5) has to be adapted to describe

the measured lifetime in a TCSPC measurement. Strong emission from pristine donor domains is expected for D18 films with a low acceptor concentration of 1%. The unbalanced D/A ratio of 99:1 gives rise to large D18 domains exceeding the diffusion length of photogenerated excitons. Therefore, we propose that the measured TCSPC lifetime of any D18 film with 1% acceptor concentration is a weighted average, consisting of two contributions according to

$$\tau_{meas} = w_D \cdot \tau_D + w_B \cdot \tau_B = w_D \cdot \frac{1}{k_r^D + k_{nr}^D} + w_B \cdot \frac{1}{k_r^D + k_{nr}^D + k_q} \quad \textbf{(S6)}$$

The weight coefficient w_D represents the emission contribution of excitons created within large domains of D18. With the domain sizes larger than the exciton diffusion length, the excitons are not able to reach a D/A interface (k_q = 0) and are forced to recombine into the ground state. The weight coefficient w_B represents the emission contribution of excitons that are created within the diffusion length of a D/A interface and have a chance to form a charge transfer state with a quenching rate k_q. Accordingly, the TCSPC lifetime of a D18:PMI-FF-PMI blend (99:1) with large domain sizes and a moderate quenching rate is expected to approach the PL lifetime of the pristine D18 film. For films with high quenching rates (D18:Y6 or D18:PC71BM), a reduction of the measured lifetime can be realized as depicted in **Figure 5**c. However, even for highly efficient D/A blends the emission of pristine D18 domains affects the measured lifetime, as indicated by the large difference between τ_{meas} and τ_{calc}.

With a similar approach, it is possible to explain the TCSPC results presented in **Figure 5**a&b. As discussed in the experimental section, the lifetimes of the D18:PMI-FF-PMI blend films with varying acceptor concentrations are found in between the lifetimes of the pristine donor and acceptor. This can be explained by the fact that the PL of the D18:PMI-FF-PMI solar cell with an optimized BHJ morphology is entirely dominated by singlet emission of pristine donor or acceptor. From the bias PL measurements in **Figure 4**b we know that the PL signal is insensitive to an applied bias (I_{SC} vs V_{OC} conditions). The minimal detectable change in PL intensity of the experimental setup is assumed to be 1%. Accordingly, the insensitivity of the bias-PL signal of D18:PMI-FF-PMI suggests that more than 99% of the emission stems from bias-insensitive singlet emission and that the radiative recombination of free charge carriers via the CT-state accounts for less than 1% of the total emission. Consequently, the measured PL lifetime in TCSPC experiments is expected to be a weighted average of the pristine donor and pristine acceptor lifetimes. In this case, the summation in equation (S6) has to be extended with an acceptor term ($w_D \cdot \tau_A$). For blends with a low quenching rate such as D18:PMI-FF-PMI, the third

term in equation (S6) does not lead to a significant reduction of the lifetime. Thus, the measured lifetime is governed by the weighted average of the pristine lifetimes of donor and acceptor, as confirmed by the experimental results in **Figure 5**a&b. Moreover, changing the detection wavelength from 650 (PMI-FF-PMI emission peak) to 700 nm (D18 emission peak) allows manipulating the weight coefficients w_D and w_A. Consequently, the lifetime of the D18:PMI-FF-PMI 1:1 blend (yellow curve in **Figure 5**a) is significantly increased compared to the pristine D18 lifetime due to the increased PMI-FF-PMI emission at a detection wavelength of 650 nm. In contrast, at a detection wavelength of 700 nm (enhanced D18 emission), the lifetimes of the 1:1 blend and the pristine D18 film are almost identical as shown in **Figure 5**b.

NoteS5_T1: Measured and calculated PL lifetime of different D/A blends. Equation (S5) was used to calculated τ_{calc} from the rates summarized in **Table 3**.

**D/A blend**	**D/A ratio**	τ_{meas} (ns)	τ_{calc} (ns)
Pristine D18	1:0	0.62	0.62
D18:PMI-FF-PMI	99:1	0.62	0.51
D18:Y6	99:1	0.35	<0.04
D18:PC_{71}BM	99:1	0.45	<0.04

Solar cell performance in % of the SQ-limit

Table S2: Measured photovoltaic parameters of D18:PMI-FF-PMI, D18:Y6, and D18:PC71BM solar cells in comparison with their respective parameters in the SQ-limit.

		V_{OC} (V)	J_{SC} (mA cm^{-2})	FF (%)	PCE (%)
**D18:PMI-FF-PMI**	measured	1.41	6.09	60.9	5.34
	SQ-limit	1.72	14.25	92.3	22.6
	% of SQ-limit	82.0%	42.7%	66.0%	23.6%
**D18:Y6**	measured	0.87	24.46	70.5	15.2
	SQ-limit	1.09	33.98	89.0	33.2
	% of SQ-limit	79.8%	72.0%	79.2%	45.6%
**D18:PC71BM**	measured	0.98	11.26	71.4	8.0
	SQ-limit	1.48	20.07	91.3	27.1
	% of SQ-limit	66.1%	56.1%	78.2%	29.3%

Charge injection model

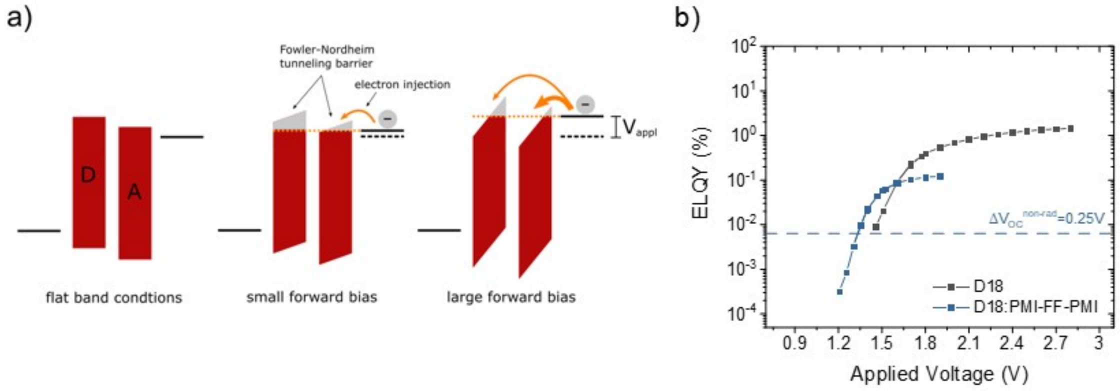

Figure S13: Charge carrier injection. a) Schematic sketch of the electron injection into the D/A blend from a high work function metal electrode. Due to the small energetic offset between the LUMO levels of donor and acceptor, the electron injection into the LUMO level of the donor is possible at large forward bias. b) ELQY measurements of pristine D18 and D18:PMI-FF-PMI 1:1 devices versus the applied voltage.

Triple junction EQE optimization potential

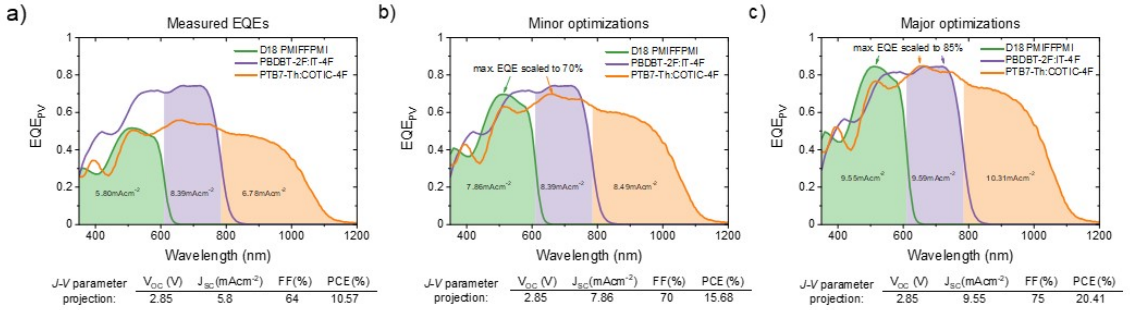

Figure S14: Optimization potential of an all-organic triple-junction solar cell. a) The measured EQE_{PV} spectra of D18:PMI-FF-PMI, PBDBT-2F:IT-4F, and PTB7-Th:COTIC-4F solar cells. The EQE_{PV} of the PTB7-Th:COTIC-4F was taken from Reference [5].

Note S6:

The single-junction solar cells D18:PMI-FF-PMI, PBDBT-2F:IT-4F, and PTB7-Th:COTIC-4F were chosen as possible candidates for an application in triple junction devices. The photovoltaic parameters of the solar cells are summarized in **NoteS6_T1**. As discussed in the introduction,

the PTB7-Th:COTIC-4F device shows strong optical absorption until approximately 1100 nm, which makes this solar cell an ideal candidate for the small-bandgap sub-cell of a triple-junction device. The EQE_{PV} of this device was extracted from the data presented in reference [5]. The EQE_{PV} of D18:PMI-FF-PMI and PBDBT-2F:IT-4F were measured with the EQE_{PV} setup described in the methods section. The single-junction EQE_{PV} spectra were used to derive a simple model to estimate the efficiency of the triple-junction device. We assume that the V_{OC} of the triple-junction device is the sum of the open-circuit voltages of the sub-cells and that the FF of the triple-junction device is the average of the observed FFs of the individual sub-cells. Furthermore, the current of the triple junction device is limited by the minimum current of the three sub-cells, which highlights the importance of "current matching" of the individual sub-cells. In our simple model, the short circuit currents of the sub-cells are calculated by integrating the product of the EQE_{PV} and the AM1.5G solar spectrum within the spectral boundaries highlighted in **Figure S14**a (shaded area under the curves). Once again it should be emphasized that this model is derived to give a rough estimate of the triple-junction device performance. Optical modeling would be necessary to incorporate interference or layer thickness effects. The model also assumes that all the light within the spectral absorption region of the front and middle sub-cells is absorbed and is not transmitted to the sub-cells below. With these assumptions, a power conversion efficiency of 10.6 % is derived for this specific triple-junction stack. According to our model an impressive V_{OC} around 2.85 V, a J_{SC} around 5.8 mAcm^{-2} and a FF around 64 % can be expected from the measured single-junction EQE_{PV} spectra. As shown in **Figure S14**a, the currents of the sub-cell are not ideally matched and lead to a serious performance decrease. The large current mismatch could be reduced by increasing the EQE_{PV} of the D18:PMI-FF-PMI and PTB7-Th:COTIC-4F sub-cells. Further improvements of wide and small-bandgap sub-cells can substantially boost the overall performance as shown in **Figure S14**b&c. Assuming a maximum EQE_{PV} of 70 % in combination with an increased average FF of 70 % significantly reduces the current mismatch and allows for PCEs around 15 %. In an optimization scenario, where the EQE_{PV} maximum of all the sub-cells is improved to 85 % and with an increased average FF of 75 %, PCEs beyond 20 % seem feasible. EQE_{PV}s of 85 % and FFs of 75 % have been reported for state-of-the-art single-junction cells and are thus considered as best-case scenarios for D18:PMI-FF-PMI and PTB7-Th:COTIC-4F sub-cell optimization.

NoteS6_T1: Measured photovoltaic parameters of D18:PMI-FF-PMI, PBDBT-2F:IT-4F and PTB7-Th:COTIC-4F solar cells. PTB7-Th:COTIC-4F data has been taken from the ESI of Reference [5].

**Material**	V_{OC} (V)	J_{SC} (mA cm^{-2})	**FF** (%)	**PCE** (%)
D18:PMI-FF-PMI	1.41	6.1	61	5.3
PBDBT-2F: IT-4F	0.87	20.1	70	12.2
PTB7-Th:COTIC-4F[5]	0.57	23.1	60	7.8

Device fabrication of solar cells in the inverted structure

Note S7:

A zinc oxide precursor solution was prepared by dissolving 0.5 g zinc acetate dihydrate in 5 mL 2-methoxyethanol and 150 µL ethanolamine followed by stirring for at least 12 h. The filtered (PVDF syringe filter, pore size: 0.45 µm) precursor solution was spin-coated onto patterned, cleaned and plasma etched glass/ITO substrates (15 × 15 × 1.1 mm^3, 15 Ω/sq, Luminescence Technology Corp.) at 4000 rpm for 30 s and afterward annealed at 150 °C for 15 minutes in air. The donor/acceptor solutions in chlorobenzene were prepared in an N_2 filled glove box with D/A weight ratios of 1/0.66, 1/1, and 1/1.3 by mixing the respective amounts of 10 mg/mL stock solutions of donor and acceptor. Before mixing, the stock solutions were stirred overnight at 70 °C. The spin coating was performed using hot solutions and substrates (85 °C). The solar cell fabrication was completed by thermal evaporation of MoO_3 (10 nm) and Ag (100 nm) layers at a pressure of $1 \cdot 10^{-5}$ mbar.

J-V characteristics were recorded under nitrogen atmosphere using a Keithley 2400 source meter unit and a LabView-based software. Illumination (100 mW/cm^2) was provided by a Dedolight DLH400 lamp, calibrated using a monocrystalline silicon WPVS reference solar cell (Fraunhofer ISE). The active area of the solar cells was defined by a shadow mask (2.65 x 2.65 mm) used for the illumination.

References

1 C. M. Cardona, W. Li, A. E. Kaifer, D. Stockdale and G. C. Bazan, Electrochemical considerations for determining absolute frontier orbital energy levels of conjugated polymers for solar cell applications, *Adv. Mater.*, 2011, **23**, 2367–2371.

2 Y. Xie, W. Wang, W. Huang, F. Lin, T. Li, S. Liu, X. Zhan, Y. Liang, C. Gao, H. Wu and Y. Cao, Assessing the energy offset at the electron donor/acceptor interface in organic solar cells through radiative efficiency measurements, *Energy Environ. Sci.*, 2019, **12**, 3556–3566.

3 S. Weber, J. Hofinger, T. Rath, M. Reinfelds, D. Pfeifer, S. M. Borisov, P. Fürk, H. Amenitsch, M. C. Scharber and G. Trimmel, Comparison of fluorene, silafluorene and carbazole as linkers in perylene monoimide based non-fullerene acceptors, *Mater. Adv.*, 2020, **1**, 2095–2106.

4 J. Hofinger, C. Putz, F. Mayr, K. Gugujonovic, D. Wielend and M. C. Scharber, Understanding the low voltage losses in high-performance non-fullerene acceptor-based organic solar cells, *Mater. Adv.*, 2021, **2**, 4291–4302.

5 X.-K. Chen, D. Qian, Y. Wang, T. Kirchartz, W. Tress, H. Yao, J. Yuan, M. Hülsbeck, M. Zhang, Y. Zou, Y. Sun, Y. Li, J. Hou, O. Inganäs, V. Coropceanu, J.-L. Bredas and F. Gao, A unified description of non-radiative voltage losses in organic solar cells, *Nat Energy*, 2021, **6**, 799–806.
